# Photoactivation of TGFβ/SMAD signaling pathway ameliorates adult hippocampal neurogenesis in Alzheimer’s disease model

**DOI:** 10.1186/s13287-021-02399-2

**Published:** 2021-06-11

**Authors:** Xiaolei Wu, Qi Shen, Zhan Zhang, Di Zhang, Ying Gu, Da Xing

**Affiliations:** 1 MOE Key Laboratory of Laser Life Science & Institute of Laser Life Science, College of Biophotonics, South China Normal University, Guangzhou, 510631 China; 2 Guangdong Provincial Key Laboratory of Laser Life Science, College of Biophotonics, South China Normal University, Guangzhou, 510631 China; 3 Department of Laser Medicine, First Medical Center of PLA General Hospital, Beijing, 100853 China

**Keywords:** Adult hippocampal neurogenesis, Photobiomodulation therapy, Neural stem cell, Newborn neuron, Alzheimer’s disease model, TGFβ/Smad pathway, Spatial learning/memory ability

## Abstract

**Background:**

Adult hippocampal neurogenesis (AHN) is restricted under the pathological conditions of neurodegenerative diseases, especially in Alzheimer’s disease (AD). The drop of AHN reduces neural circuit plasticity, resulting in the decrease of the generation of newborn neurons in dentate gyrus (DG), which makes it difficult to recover from learning/memory dysfunction in AD, therefore, it is imperative to find a therapeutic strategy to promote neurogenesis and clarify its underlying mechanism involved.

**Methods:**

Amyloid precursor protein/presenilin 1 (APP/PS1) mice were treated with photobiomodulation therapy (PBMT) for 0.1 mW/mm^2^ per day in the dark for 1 month (10 min for each day). The neural stem cells (NSCs) were isolated from hippocampus of APP/PS1 transgenic mice at E14, and the cells were treated with PBMT for 0.667 mW/mm^2^ in the dark (5 min for each time).

**Results:**

In this study, photobiomodulation therapy (PBMT) is found to promote AHN in APP/PS1 mice. The latent transforming growth factor-β1 (LTGFβ1) was activated in vitro and in vivo during PBMT-induced AHN, which promoted the differentiation of hippocampal APP/PS1 NSCs into newborn neurons. In particular, behavioral experiments showed that PBMT enhanced the spatial learning/memory ability of APP/PS1 mice. Mechanistically, PBMT-stimulated reactive oxygen species (ROS) activates TGFβ/Smad signaling pathway to increase the interaction of the transcription factors Smad2/3 with Smad4 and competitively reduce the association of Smad1/5/9 with Smad4, thereby significantly upregulating the expression of doublecortin (Dcx)/neuronal class-III β-tubulin (Tuj1) and downregulating the expression of glial fibrillary acidic protein (GFAP). These in vitro effects were abrogated when eliminating ROS. Furthermore, specific inhibition of TGFβ receptor I (TGFβR I) attenuates the DNA-binding efficiency of Smad2/3 to the Dcx promotor triggered by PBMT.

**Conclusion:**

Our study demonstrates that PBMT, as a viable therapeutic strategy, directs the adult hippocampal APP/PS1 NSCs differentiate towards neurons, which has great potential value for ameliorating the drop of AHN in Alzheimer’s disease mice.

**Supplementary Information:**

The online version contains supplementary material available at 10.1186/s13287-021-02399-2.

## Background

In the adult mammalian brain, neural stem cells (NSCs) are mainly identified in two major regions: the subgranular zone (SGZ) of the hippocampus and the subventricular zone (SVZ) of the lateral ventricle [1, 2]. The hippocampus, a brain area critical for learning and memory and one of the first brain regions to be affected in Alzheimer’s disease (AD), contains NSCs that continue to generate new neurons throughout life, a process called adult hippocampal neurogenesis (AHN) [3, 4]. Newborn neurons that are generated from NSCs residing in the SGZ are constantly migrated to the granule cell layer (GCL) and integrated into the existing hippocampal neural circuitry [5] sustain the generation of young neurons throughout life in mice, which is required for brain homeostasis and is implicated in learning/memory and affective behaviors [6–8]. Some neurodegenerative diseases, including AD, Parkinson’s disease (PD), and Huntington’s disease (HD), have been associated with restrictions in AHN [7, 9, 10].

AD is a neurodegenerative disease, and its pathogenesis includes brain atrophy, amyloid-β (Aβ) plaque deposit [11], tau protein aggregates in the form of intracellular neurofibrillary tangles (NFTs), and other pathological changes that cause the formation of senile plaque (SP) [12, 13]. The hippocampus is one of the most affected brain regions in AD [14], and the dentate granule cells (DGCs) exhibit remarkable morphological alterations in patients with AD [15]. Increasing AHN is sufficient to improve in hippocampus-dependent learning [16], however, AHN drops sharply in various AD mouse models, raising the possibility that reduced AHN causes memory impairments and cognitive deterioration in this disease [17–19]. In recent research, some progress has been made in using exogenous pathways and allogeneic transplantations of NSCs/induced pluripotent stem cells (iPSCs) to promote neurogenesis for the treatment of neurodegenerative diseases [20]. Furthermore, learning/memory deficits caused by reduced neurogenesis have been shown to be rescued by the transplantation of hilar inhibitory interneurons [3, 21]. These findings imply that promotion of neurogenesis may provide a feasible treatment to ameliorate the progression of AD.

An increasing number of experimental and clinical studies have shown that photobiomodulation therapy (PBMT) has been demonstrated to be an appealing modality for regenerative medicine, such as in promoting regeneration in hair, lung, skin, and regulating the neuronal function [22–28] , but whether PBMT can regulate the proliferation and differentiation of NSCs in AD is still unclear. Importantly, Arany et al. demonstrated that photoactivation of endogenous latent transforming growth factor-β1 (LTGFβ1) directs dental stem cell differentiation for regeneration [29]. Proteins in the TGFβ superfamily bind to cell-surface receptors and subsequently phosphorylate the intracellular effector, Smad, at two C-terminal serines, and receptor-activated Smads (R-Smads) are then associated with one co-Smad, namely, Samd4 [30]. These trimeric complexes translocate into the nucleus, after which they act as transcription factors to regulate the expression of target genes [31]. Transforming growth factor-β1-3 (TGFβ1-3) are broadly and dynamically expressed in the developing central nervous system (CNS) [32], where they regulate temporal identity and potency of NSCs; furthermore, these proteins can be applied to modulate temporal specification of neurons via stem cell engineering [33]. Inhibiting TGFβ receptor I (TGFβR I) increases expression levels of both Nanog and Octamer-binding transcription factor 4 (Oct4), after which there is an absence of expression of lineage-differentiation markers [34, 35]. Such properties support that TGFβ signaling may be a key player in stem cell pluripotency and differentiation. In addition, our previous studies have shown that PBMT decrease neuron loss, ameliorate dendritic atrophy, and provide overall neurodegenerative disease improvement [25–28]. These findings suggest that PBMT, with efficacious neurobiological protective effects, possibly be used as an intervention to promote AHN by transduction of neurogenic-niche LTGFβ1 signals to trigger a signaling cascade that activates intracellular regulatory mechanisms in amyloid precursor protein/presenilin 1 (APP/PS1) NSCs.

## Methods

### Experimental design

For in vivo experiment, wild-type (WT) mice were randomly divided into WT group and WT + PBMT group, and APP/PS1 double transgenic mice were randomly divided into APP/PS1 group and APP/PS1 + PBMT group. The WT + PBMT group and APP/PS1 + PBMT group mice were treated with PBMT (0.1 mW/mm^2^) for 10 min per day for 1 month, and the irrelevant variables in the experiment were consistent until the end of the experiment. No other replication studies were performed. No statistical method was used to determine the sample size before the experiment, and no data or animals were excluded from analysis. For in vitro experiment, WT NSCs and APP/PS1 NSCs were randomly divided into control group, PBMT group, N-acetyl cysteine (NAC) + PBMT group, SB431542 + PBMT group, NAC group, SB431542 group, and LDN193189 group, respectively. The groups of PBMT, NAC + PBMT, and SB431542 + PBMT were treated with PBMT (0.667 mW/mm^2^) for 5 min each time. NAC (1 mM) was added 0.5 h before PBMT in the NAC + PBMT group, and SB431542 (60 μM) was added 0.5 h before PBMT in the SB431542 + PBMT group. Correspondingly, reagents corresponding to each group were added in the NAC group, SB431542 group, and LDN193189 (0.5 μM) group respectively. And in this study, a variety of assays were used in vivo or in vitro to assess key points in neurogenesis, including expression of newborn neuron-associated proteins and improvement in learning or cognitive impairment in APP/PS1 transgenic mice. For in vitro or in vivo experiments, sample sizes are described in the figure legends.

### Animals

The transgenic mice (APP/PS1) used in this study were purchased from the Jackson Laboratory, the characterization of the APP/PS1 double transgenic mice, expressing a chimeric mouse/human amyloid precursor protein bearing the Swedish mutation (Mo/HuAPP695swe) and a mutant human Presenilin 1 protein (PS1-dE9) in central nervous system neurons. Mouse genotypes were determined by polymerase chain reaction (PCR) analysis of tail biopsies. To more accurately mimic the clinical setting, we used 6-month-old male APP/PS1 mice with a preexisting subset of the behavioral and pathological features of AD and their non-transgenic wild-type (WT) littermates as control group [28]. WT and transgenic mice were housed under the same living conditions (at a constant temperature of 22 + 1 °C, humidity of 50–60%, and lights on from 07:00 to 19:00. All of the mice were provided food and water ad libitum).

Mice were housed in the animal facilities at the Institute of Biophotonics Research at South China Normal University. All of the animal procedures and breeding were approved by the Institutional Animal Care and Use Committee of the South China Normal University (Number: SCNU-BIP-2021-018). All animal experiments followed the guidelines of The Care and Use of Laboratory Animals (Institute of Laboratory Animal Resources, Commission on Life Sciences, National Research Council). For the experiments involved in the current study, a total of 48 6-month-old APP/PS1 mice were randomly divided into APP/PS1 group, APP/PS1 + PBMT group, with 24 mice in each group. In addition, 48 6-month-old C57BL6J mice were randomly divided into WT group and WT + PBMT group with 24 mice in each group. Only males were used for this study to reduce gender-specific variability in the behavioral test. At the end of the study, mice were sacrificed, and the brain tissue was harvested [36]. For the isolation of neural stem cells, we typically harvest pups at E14, sacrificing the pregnant mouse in accordance with rules dictated by the animal ethics committee.

### Behavioral test

#### Y maze test

For measurement of spontaneous alternation in the Y maze, each mouse was placed at the end of an arm and was then allowed to enter the maze. When all of the paws of the mouse entered the arm, a camera was used to record the order in which the mouse moved into each arm within 7 min of free movement. Each mouse was continuously recorded for 3 days, and the mouse was placed in different arms each time. Measurement of spatial memory was achieved using the Y maze with a blocked arm. In the period of acquisition, one arm was closed and the mouse was allowed to freely explore for 3 min in the other arm. During this time, we recorded the number of entries in each arm and the time and distance of exploration in each arm. We also recorded the recall period after 2 h; at this point, all of the arms were opened and the mouse was free to explore for 3 min among the three arms (start arm, novel arm, and the other arm). We recorded the time and distance of exploration in each arm, recorded for three consecutive days, and closed different arms each time during the acquisition period. In the entire Y maze test, all of the laboratory instruments and objects were washed with 75% ethanol before and after each experiment.

#### Morris water maze (MWM) test

Last week during PBMT treatment, hippocampus-dependent spatial learning and memory abilities were evaluated with the MWM as previously described methodology [37, 38]. Briefly, the apparatus consists of a round metal pool (100 cm in diameter) filled with opaque water at 22 ± 0.5 °C. The maze is divided into four quadrants, one of which has a clear escape platform with a diameter of 10 cm located 1.0 cm below the surface of the water. The room was well lit and there were obvious clues in the walls. First, the mice were trained to find the escape platform with the help of the maze cues for 1 day. During the acquisition time, the mice were given four trials for 5 days. They were randomly placed in the water. The escape time of finding the platform and climbing onto it was recorded. If the mice could not find the platform in the allotted time (60 s), the mice were placed onto it for 10 s manually. The spatial probe test was performed after the removal of the platform on the 6th day, and the mice were allowed to swim for 60 s to determine their search bias.

### PBMT treatments

PBMT treatments were conducted as described in our previous work [25–27]; LTGFβ1 was added to the culture medium at 5 min before PBMT treatment. The cells were irradiated with a semiconductor laser (635 nm, NL-FBA-2.0-635, nLight Photonics Corporation, Vancouver, WA, USA) for 5 min in the dark, with a corresponding fluence of 2 J/cm^2^. In each experiment, the surrounding environment was kept completely dark or very dim in order to minimize any environmental interference from light. In animal experiments, 6-month-old WT and APP/PS1 mice (eight mice per group) were treated with PBMT for 10 min per day for 1 month. In this study, the laser power density propagated through the transparent bag/fresh scalp/skull penetration rate was calculated, and the transmittance of 635 nm PBMT penetrating the scalp and skull to the interior of the hippocampus was approximately 33% [28]. Therefore, the energy density corresponding to the cerebral cortex tissue level was adjusted to 6 J/cm^2^, without any local temperature increase in the scalp and brain tissue of mice during irradiation. The calculation formula of designated time for PBMT treatment is time (s) = energy (J/cm^2^) × surface (cm^2^)/power (W) [26]. For details of laser parameters and treatment parameters used in PBMT treatment in vivo and in vitro, see Tables S2–S3.

### Isolation of neural stem cells (NSCs)

The NSCs used in this study were isolated from hippocampus of C57BL/6J and APP/PS1 transgenic mice at E14. In detail, in order to establish embryonic neurosphere culture, we referred to methods already described in previous literature [39–42], typically harvest pups at E14, anesthetize the pregnant mouse by an intraperitoneal injection of pentobarbital (120 mg/kg), and on deep anesthesia, sacrifice it by cervical dislocation. We lay the pregnant mouse on its back on the absorbent towels, and then liberally rinse the abdomen with 75% ethanol so as to sterilize the area, remove the uteri with small forceps and scissors, and transfer them into a 10-cm dish containing HBSS. We transfer uterine tissues to a 10-cm Petri dish containing fresh sterile HBSS. We cut open the uterine horns and transfer the pups to a new 10-cm dish containing HBSS, using small forceps. We separate the head(s) of the pup(s) at the level just below the cervical spinal cord, discarding the skulls. We transfer each tissue culture dish to a dissecting microscope, dissect out the hippocampus to be used for establishing the culture, and transfer each harvested brain region to a 1.5-ml centrifuge tube containing 1-ml ice-cold HBSS. We purge the obtained tissue twice and discard the HBSS, then, add 0.05% trypsin to the tissue, digest at 37 °C for 10 min, add DMEM/F12 containing 10% serum to stop the digestion, discard the waste tissue after blowing several times, and centrifuge the culture medium containing the cells at 1800 rpm for 8 min, and precipitation is NSCs. NSCs were maintained in DMEM/F-12 medium containing 20 ng/ml of basic fibroblast growth factor (bFGF), 20 ng/ml of epidermal growth factor (EGF), and 2% B27. We use Nestin antibody for cell immunofluorescence to identify NSCs, and almost all neurospheres are Nestin positive [39]; when the neurospheres proliferated by 3–4 days in culture, they were digested with StemPro Auccutase and were then seeded in a plate previously coated with 0.6% Matrigel for at least 1 h at 37 °C. The cells were cultured in an incubator at 5% CO_2_ and 37 °C and half of the medium was replaced every 2 days.

### ELISAs for LTGFβ1 activation

We used the ELISA Emax immunoassay kit (Promega) to evaluate the activation of exogenously added LTGFβ1 in the NSC culture medium after laser irradiation. LTGFβ1 (2.5 ng/ml) was added to the cell culture medium at 5 min, and some groups were preincubated with NAC (1 mM) at 1 h before the laser irradiation. After irradiation, cells were cultured in an incubator for 30 min, after which the supernatants of the cells were collected under the same conditions and diluted by 1:50 for viability assays. All of the experimental procedures were performed according to the corresponding manufacturer’s instructions.

### Western blotting and co-immunoprecipitation

Brain tissues and cells were lysed on ice for 60 min with lysis buffer (50 mM Tris-HCl, pH 8.0, 150 mM NaCl, 1% TritonX-100, 100 μg/mL PMSF) containing Complete Protease Inhibitor Cocktail, and the lysates were then centrifuged at 12,000 rpm for 15 min. Supernatants were separated on a sodium-dodecyl sulfate polyacrylamide gel (SDS-PAGE) and were blotted with the indicated antibodies (described in Table S1 of Supplemental materials). For co-immunoprecipitation, protein extracts were incubated with the indicated antibodies for 2–4 h at room temperature and were then incubated overnight with 50% slurry protein A-Sepharose at 4 °C. Each pellet was resuspended in an equal volume of SDS sample buffer, boiled for 8 min, centrifuged at 12,000 rpm for 30 s to remove protein A-Sepharose beads, and then analyzed by Western blotting for whole-cell lysates and immunoprecipitates. The immunoblotting results were analyzed by Odyssey and ImageJ software.

### Immunocytochemistry

After treatments, the cells were fixed with 4% paraformaldehyde (PFA), permeabilized with 0.5% Triton X-100, and blocked with 5% bovine serum albumin. These steps were performed at room temperature. After blocking, the cells were incubated with primary antibodies at 4 °C overnight and were then incubated with immunofluorescent secondary antibodies for 2 h at room temperature. After sealing, the cells were analyzed by confocal microscopy (LSM880 META; ZEN black) and LSM880 META software (ZEN blue). Primary antibodies, secondary antibodies, and their dilutions are provided in the Tables S1.

### Tissue preparation and immunohistochemistry

Mice were euthanized by intraperitoneal injection of avertin and were then transcardially perfused with saline followed by 4% PFA. Brains were dissected, post-fixed overnight in 4% PFA and then equilibrated in 15% and 30% sucrose. Subsequently, brains were embedded with OCT and sectioned in 10-μm-thick slices. Tissue sections were fixed with 4% PFA, permeabilized with 0.5% Triton X-100, and blocked with 5% bovine serum albumin. Primary antibodies were visualized with Alexa-conjugated secondary antibodies. Primary antibodies, secondary antibodies, and their dilutions are provided in the Tables S1. After staining, sections were mounted, coverslipped, and maintained at 4 °C in the dark until further analysis. Confocal single-plane images were taken with a Zeiss LSM 880 microscope. Images were processed using ZEN blue and ImageJ software.

### Quantitative real-time PCR (RT-qPCR)

Total RNA was isolated using RNA iplus (TaKara, 91090) based on the manufacturer’s instructions. The first strand of cDNA was generated using a reverse transcription kit, and the target gene was amplified using gene-specific primers and SYBRGreen PCR Mix via a Bio-Rad real-time PCR system to quantify mRNA levels in real time. The amount of cDNA in the PCR reaction system was 800 ng, the concentrations of the upstream and downstream primers were 300 nM, and the volume of the final reaction system was 20 μl. The results of different samples were calculated by Bio-Rad’s built-in data analysis software. The primer sequences are shown in the Tables S1.

### Chromatin immunoprecipitation assay

The ChIP experiment was performed according to the procedure in the ChIP experimental kit (CST, 9003) instructions. Primary NSCs of each treatment group were cross-linked with 1% formaldehyde at room temperature for 10 min, and the fixation reaction was terminated by incubation with glycine at room temperature for 5 min. Samples were rinsed twice with ice-cold PBS, and the supernatant was removed immediately after nuclear processing and chromatin cleavage. Next, the 0.3 μl/4 × 10^6^ cells of microsphere nuclease were added to the lysate, after which the mixture was inverted and mixed several times, incubated at 37 °C for 20 min, and mixed once every 3–5 min to digest DNA fragments that were 150–900 bp in length. Next, a Smad2/3 antibody was added to each sample tube, and IgG was added to each group of negative control tubes. The reaction tubes were then sealed and incubated at 4 °C overnight, and then each immunoprecipitation reaction was mixed with 30 μl of ChIP-grade protein G magnetic beads and incubated for 2 h at 4 °C to form antibody/DNA/magnetic-bead complexes. Next, 150 μl of elution buffer was added to each ChIP immunoprecipitated sample, the chromosome was eluted from antibody/protein G beads and decross-linked, and the DNA was purified by a spin column to recover the DNA fragment. Analysis of ChIP enrichment efficiency was achieved via RT-PCR. The positive control consisted of histone H3 samples, whereas the negative control group consisted of normal rabbit IgG samples. The primer sequences are shown in Fig. S5.

### SB431542, NAC, and LDN193189 administrations

Systemic administrations of SB431542 (MCE, HY-10431, 60 μM), NAC (Sigma, A9165, 1 mM), and LDN193189 (MCE, HY-12071A, 0.5 μM) were performed 0.5 h before each PBMT treatment.

### Detection of reactive oxygen species (ROS) in NSCs

The NSCs were divided into different experimental groups. NAC (1 mM) was added to the cells at 1 h before treatment. At 0.5 h later, 40 μM of DCF (10 mM of mother liquor) was added. After PBMT, cells were cultured in an incubator for 15 min and were then imaged via laser confocal microscopy.

### Quantification and statistical analysis

#### Cell quantification

For quantification of cells expressing stage-specific markers, we randomly selected one of the brain tissue sections to be stained by immunofluorescence, and at least three brain sections of different mice were repeated for staining. The quantification of the stage-specific markers in the cell experiments were also repeated in at least three different mouse NSCs. The number of positive cells was counted using ImageJ. The samples sizes are described in the figure legends.

#### Statistical analysis

Statistical analyses were performed using GraphPad and Origin 6.0. Two-way analysis of variance (ANOVA) was used for escape latency analysis. The other comparisons between two or among more groups were performed with Student’s unpaired *t* tests, two-way ANOVA, and one-way ANOVA. Data were presented as the mean ± standard error of the mean (SEM), and *P* < 0.05 was considered statistically significant (see each figure for details). The sample sizes can be found in the figure legends.

## Results

### Effects of PBMT on neurogenesis in the adult hippocampus of APP/PS1 mice

**Fig. 1:**
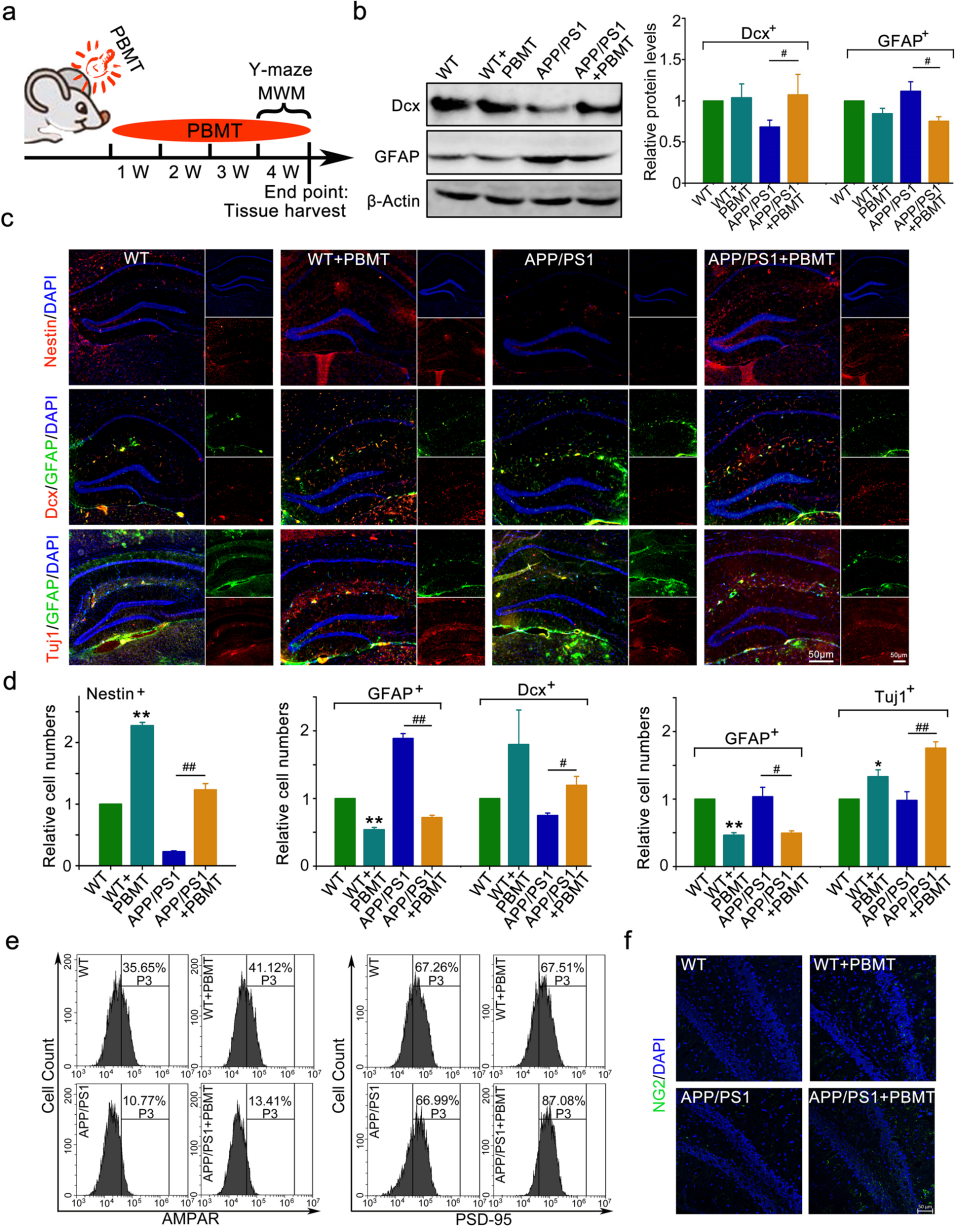
PBMT promotes both neurogenesis and gliogenesis in the adult hippocampus of amyloid precursor protein/presenilin 1 (APP/PS1) mice. **a** The experimental procedure, 6-month-old wild-type (WT) and APP/PS1 mice received photobiomodulation therapy (PBMT) treatment. **b** Western blotting analysis and quantification of doublecortin (Dcx) and glial fibrillary acidic protein (GFAP) expression from APP/PS1 transgenic mice and age matched WT mice hippocampus at 7 months of age after PBMT, (*n* = 4 per group). **c** Representative images of Nestin^+^ (neural stem cell staining), Dcx^+^ (newborn neuron staining), GFAP^+^ ( astrocyte staining), and Tuj1^+^ (newborn neuron staining) expression cells in the hippocampal dentate gyrus (DG) of WT and APP/PS1 transgenic mice after PBMT. Scale bars, 50 μm. **d** Quantification of the relative numbers of Nestin^+^, Dcx^+^, GFAP^+^, and Tuj1^+^ cells in the hippocampal DG of WT and APP/PS1 transgenic mice after PBMT (*n* = 6 per group). **e** Staining the newborn neurons with neuronal class-III β-tubulin (Tuj1) antibody, and then the flow cytometry was used to detect the expression of α-amino-3-hydroxy-5-methyl-4-isoxazole-propionic acid receptors (AMPAR) and postsynaptic density protein 95 (PSD-95) on Tuj1^+^ newborn neurons. **f** Representative immunofluorescence image of gliogenesis in vivo, using NG2 antibody staining oligodendrocyte precursor cell. All quantifications are presented as mean ± SEM and were analyzed by one-way ANOVA test; **p < 0.01, *p < 0.05 versus control group; ^##^*p* < 0.01, ^#^*p* < 0.05 versus indicated group

According to the experimental procedure (Fig. 1a), we showed that compared with that of the control group, PBMT-treated APP/PS1 mice exhibited the upregulation of doublecortin (Dcx) expression by 32% (Fig. 1b, *p* < 0.05), downregulation of glial fibrillary acidic protein (GFAP) expression by 28% (Fig. 1b, *p* < 0.05), and increased the 98% numbers of Nestin^+^ radial glial cells and 31% Dcx^+^/45% Tuj1^+^ immature neurons in the SGZ of the hippocampus (Fig. 1c, d, *p* < 0.01), while the numbers of GFAP^+^ astrocytes were relatively reduced by 85% (Fig. 1c, d, *p* < 0.01).

The results also showed that the α-amino-3-hydroxy-5-methyl-4-isoxazole-propionic acid receptors (AMPARs) and postsynaptic density protein 95 (PSD-95) on newborn neurons in APP/PS1 mice brain tissue were increased after PBMT (Fig. 1e). Moreover, the gliogenesis of APP/PS1 mice was promoted after 1 month of PBMT (Fig. 1f).

### PBMT improves spontaneous alternation and spatial learning/memory in APP/PS1 mice

**Fig. 2:**
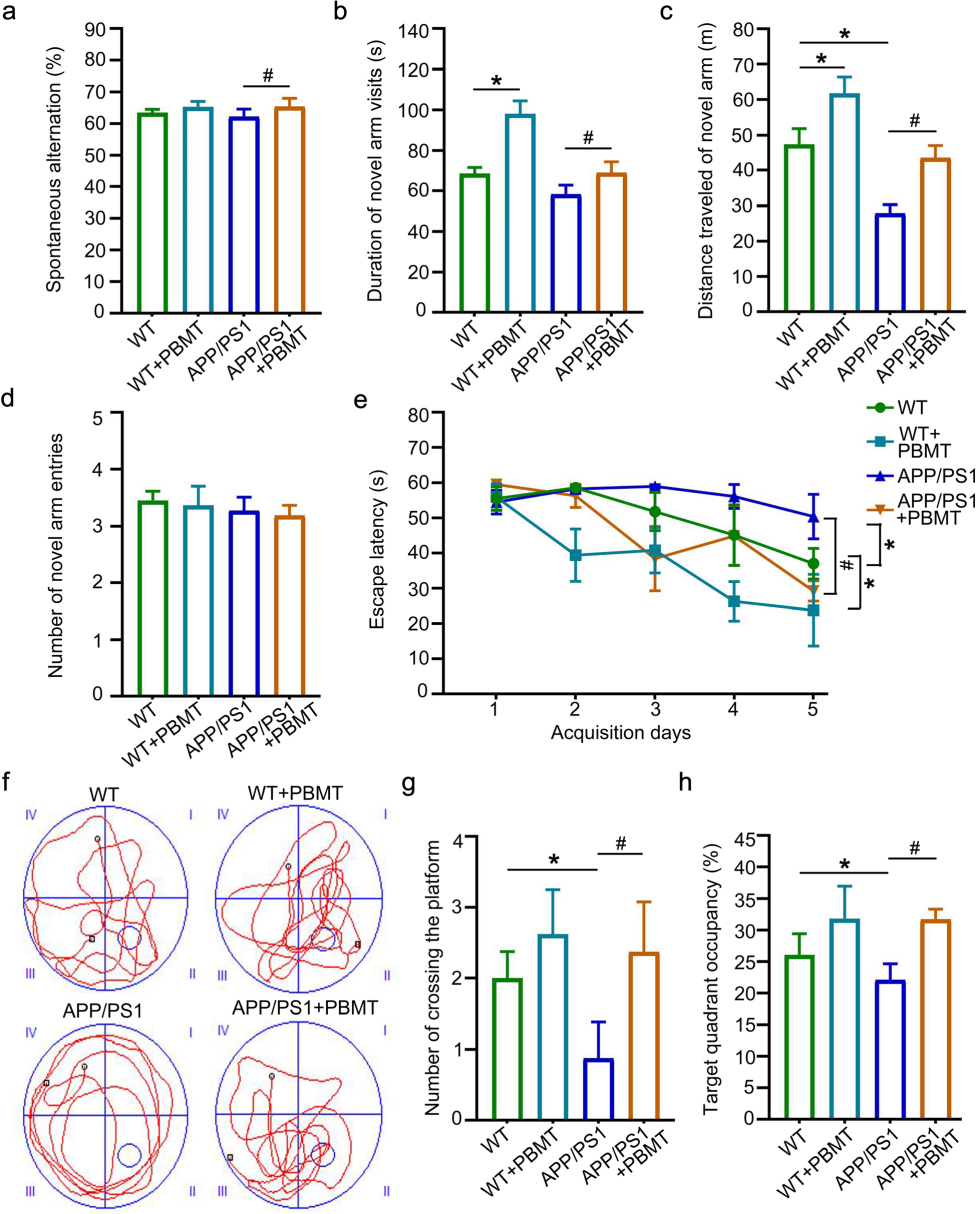
PBMT improves spatial learning/memory of APP/PS1 mice in the Y maze and Morris water maze (MWM)**. a** Spontaneous alternating data statistics after PBMT, (*n* = 8 mouse at 7 months of age for each group after PBMT). **b–d** Duration of novel arm visits data statistics (**b**), distance traveled of novel arm data statistics (**c**) and the number of novel arm entries data statistics (**d**) after PBMT (*n* = 8 mouse at 7 months of age for each group after PBMT). **e** The escape latency during the 5 days of the training phase in mice. **f** Representative swimming traces of the four groups of mice exhibited during the MWM test. **g**, **h** The number of passes across the escape platform (**g**) and the percentage of time spent in the target quadrant (**h**) during the MWM test, (*n* = 6 mouse at 7 months of age for each group after PBMT). All data were detected by the Y maze test or MWM test. All quantifications are presented as mean ± SEM and were analyzed by one-way ANOVA test; *p < 0.05, ^#^*p* < 0.05 versus indicated group

Next, we performed Y maze and Morris water maze (MWM) [43, 44] tasks to evaluate the effects of PBMT on spatial learning and memory in APP/PS1 mice. There were significant changes in spontaneous alternation by 8% (Fig. 2a, *p* < 0.05); the duration of the novel arm increased 18 s (Fig. 2b, *p* < 0.05), the distance traveled of novel arm increased 17 m (Fig. 2c, *p* < 0.05), and the number of novel arm entries have no significant changes (Fig. 2d) in mice after 1 month of PBMT treatment compared with those of the control group. And the MWM results showed that the escape latency to find the hidden platform was significantly reduced in the acquisition phase, and PBMT-treated mice significantly shortened the latency by 15 s of WT mice and 22 s of APP/PS1 mice compared with the controls (Fig. 2e, *p* < 0.05). In the probe trial phase, the PBMT-treated mice performed better than the controls (WT and APP/PS1 mice) (Fig. 2f), as reflected by the significantly 2 greater numbers of passes across the former platform (Fig. 2g, *p* < 0.05) and the 10% longer time spent in the target quadrant (Fig. 2h, *p* < 0.05).

### PBMT directs the differentiation of APP/PS1 NSCs towards neurons in vitro

**Fig. 3:**
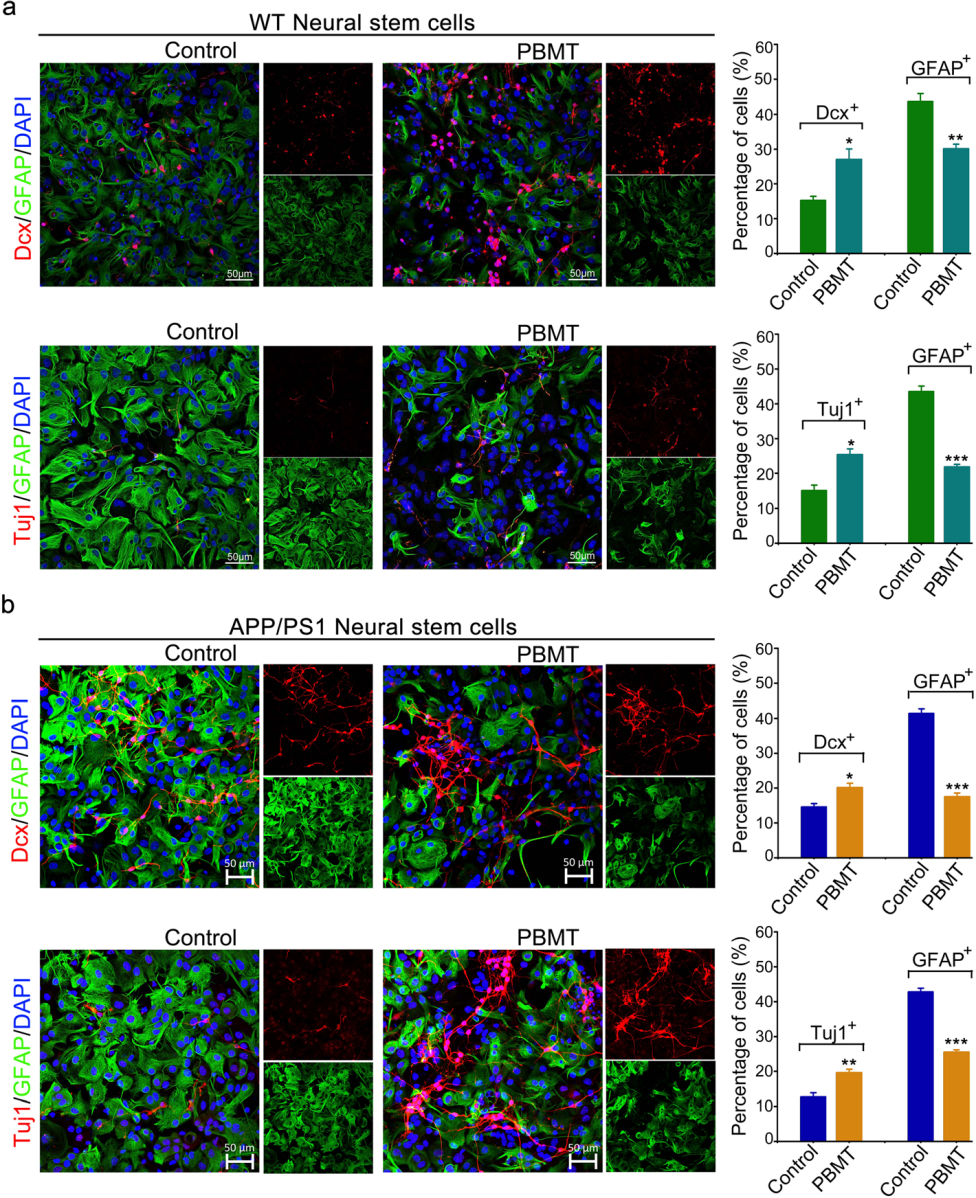
PBMT directs the APP/PS1 neural stem cells (NSCs) differentiate into neurons in vitro**. a** Immunostaining the dual differentiation capacity of WT NSCs after PBMT. Neurons and astrocytes were induced by in vitro differentiation of neurospheres isolated from WT mice DG. Scale bar, 50 μm, (*n* = 4 per group). **b** Immunostaining the dual differentiation capacity of APP/PS1 NSCs after PBMT. Neurons and astrocytes were induced by in vitro differentiation of neurospheres isolated from APP/PS1 mice DG. Scale bar, 50 μm, (*n* = 4 per group). All quantifications are presented as mean ± SEM and were analyzed by two-way ANOVA test; ***p < 0.001, **p < 0.01,*p < 0.05 versus corresponding control group

We extracted and cultured NSCs from the hippocampus of WT and APP/PS1 fetal mice (at embryonic day 14 [E14]). Matrigel was used as a supporting substrate to induce NSCs adherence (Fig. S1a, S1b ) after 3–4 days of suspension culture, and PBMT treatments were administered at 7–14 days after adherent cultures were established. Interestingly, western blotting results showed that compared with those of the control group, PBMT-treated NSCs exhibited the upregulation of Dcx expression by 15% (Fig. S1c, *p* < 0.01) and the downregulation of GFAP expression by 20% (Fig. S1d, *p* < 0.01). And immunofluorescence results revealed that PBMT increased the number of Dcx^+^ by 13% in WT NSCs (Fig. 3a, *p* < 0.05) and by 9% in APP/PS1 NSCs (Fig. 3b, *p* < 0.05), increased Tuj1^+^ cells by 10% in WT NSCs (Fig. 3a, *p* < 0.05) and by 9% in APP/PS1 NSCs (Fig. 3b, *p* < 0.01); meanwhile, PBMT decreased the number of GFAP^+^ cells by 16% in WT NSCs (Fig. 3a, *p* < 0.01) and by 20% in APP/PS1 NSCs (Fig. 3b, *p* < 0.001). Moreover, the results of the expression levels of AMPARs and microtubule-associated protein 2 (MAP2) in newborn neurons indicated that newborn neurons in both the control group and the PBMT group exhibited normal expression of relevant functional proteins that are typical in neurons (Fig. S1e, S1f).

### TGFβ1 plays a crucial role in APP/PS1 NSC differentiation and PBMT activates LTGFβ1 in vivo

**Fig. 4:**
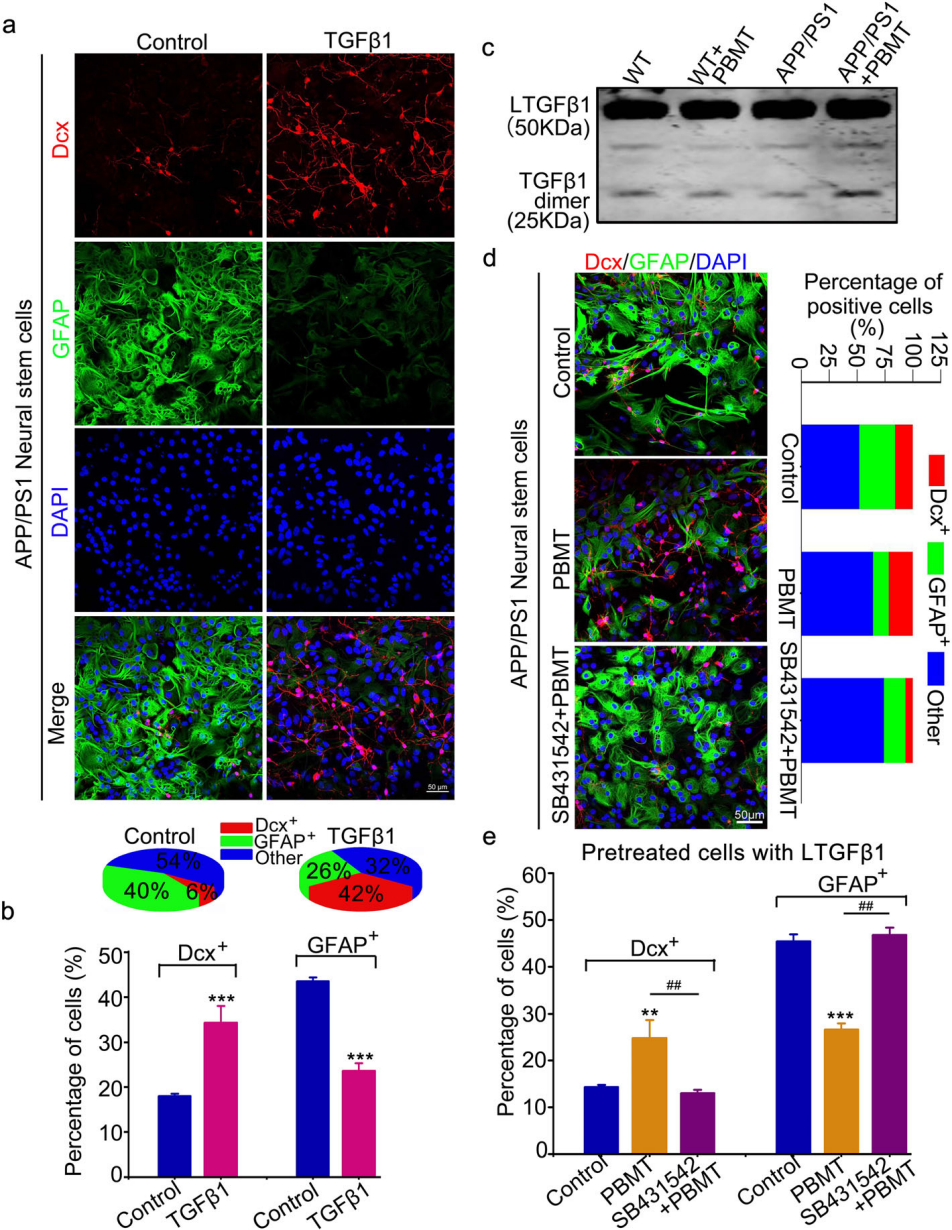
TGFβ1 plays the key role in APP/PS1 NSC differentiation and PBMT activates latent TGFβ1 (LTGFβ1) in vivo**. a**, **b** Confocal representative images of Dcx^+^ and GFAP^+^ cells (**a**) and quantification the number of Dcx^+^ and GFAP^+^ cells (**b**) after exogenously added active TGFβ1 (2 ng/ml) to induce differentiation of APP/PS1 NSCs. Scale bar, 50 μm, (*n* = 4 per group). **c** Western blotting analysis of the LTGFβ1 activation of WT and APP/PS1 transgenic mice brain hippocampus after PBMT. **d** The differentiation of APP/PS1 NSCs after PBMT, some cells were pretreated with SB431542 (TGFβR I inhibitor, 60 μM) before PBMT. Scale bar, 50 μm. **e** The quantification of Dcx^+^ and GFAP^+^ cells after PBMT, some cells were pretreated with SB431542 before PBMT (*n* = 4 per group). All quantifications are presented as mean ± SEM and were analyzed by one-way ANOVA test; ***p < 0.001, **p < 0.01 versus control group; ^##^*p* < 0.01 versus indicated group

To explore whether TGFβ1 has a direct effect on the differentiation of APP/PS1 NSCs into neurons, we exogenously replenished TGFβ1 (2 ng/ml) during 7–14 days of in vitro APP/PS1 NSCs differentiation culture, after which we detected the number of Dcx^+^/GFAP^+^ cells via cellular immunofluorescence. The results showed that TGFβ1 increased the number of Dcx^+^ cells by 16% and reduced the number of GFAP^+^ cells by 18% compared with those in control group NSCs (Fig. 4a, b, *p* < 0.001). In order to explore whether the results of in vivo and in vitro mechanisms are consistent, we also detected the activation of LTGFβ1 in vivo after PBMT via western blotting assays. The western blotting results showed that the LTGFβ1 was activated by PBMT in APP/PS1 mice (Fig. 4c). Importantly, we preincubated cells with LTGFβ1 and an TGFβRI inhibitor (SB431542) before PBMT and detected the number of Dcx^+^ and GFAP^+^ cells after differentiation of APP/PS1 NSCs for 14 days. Similarly, we found that pretreated cells with LTGFβ1 before PBMT increased the differentiation of APP/PS1 NSCs into neurons by 12% (Fig. 4d, e, *p* < 0.01) and reduced the differentiation into astrocytes by 19% (Fig. 4d, e, *p* < 0.001), but SB431542 suppressed the effect of PBMT on APP/PS1 NSCs (Fig. 4d, e, *p* < 0.01). Collectively, these findings suggest a major role for TGFβ1 in mediating PBMT-induced AHN.

### The generation of ROS in NSCs activates LTGFβ1 to direct the APP/PS1 NSC differentiation via in vitro-induced PBMT

**Fig. 5:**
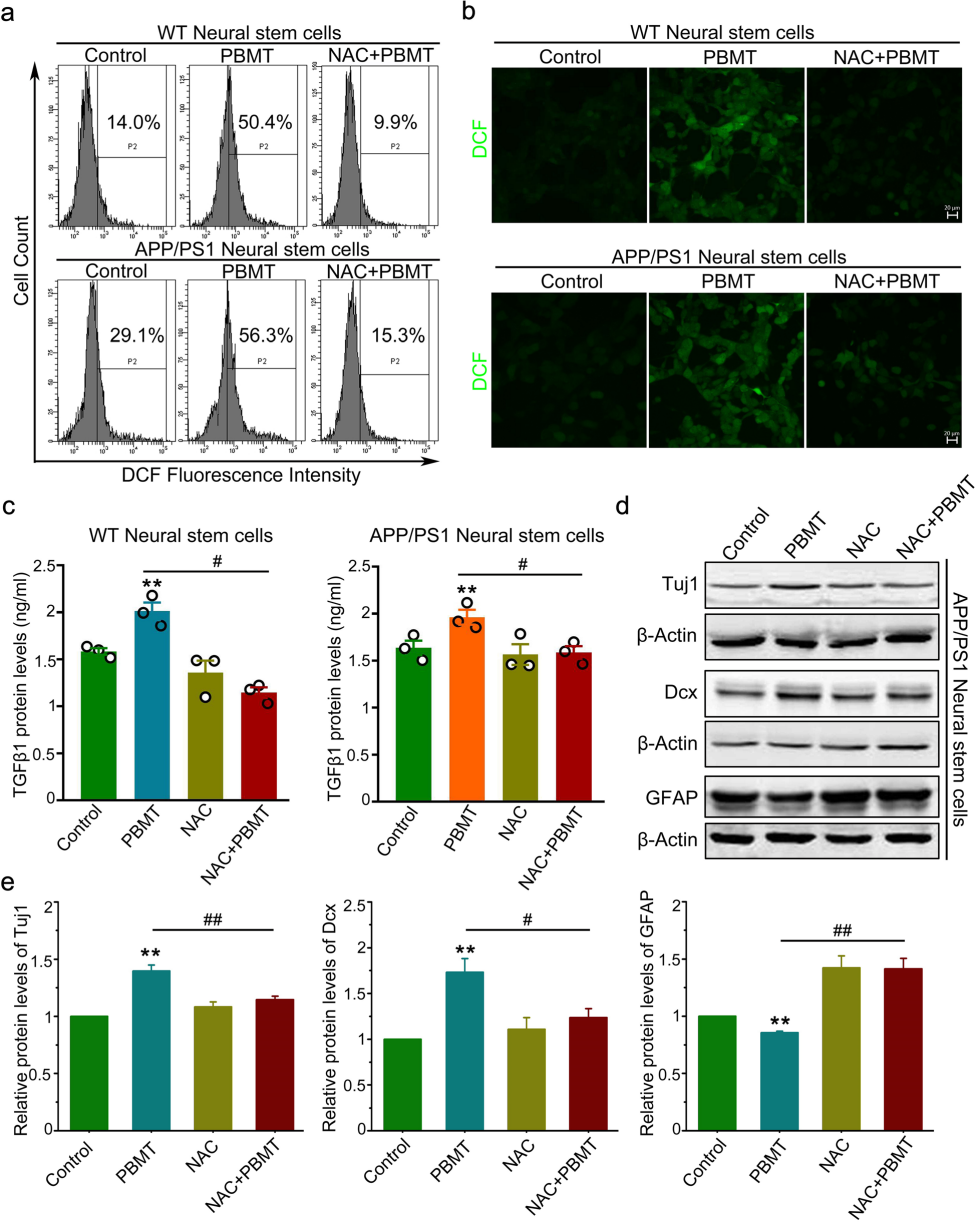
PBMT-induced reactive oxygen species (ROS) generation in NSCs activates LTGFβ1 to direct the NSC differentiation**. a, b** Flow cytometry (**a**) and confocal microscopy (**b**) detect ROS generation (revealed by DCFH-DA) of WT and APP/PS1 NSCs after PBMT. Some cells were preincubated with N-acetyl cysteine (NAC, 1 mM) before PBMT. Scale bar, 20 μm. **c** Enzyme-linked immunosorbent assays (ELISA) assess the activated TGFβ1 by ROS, isolation and culture of NSCs from hippocampus of WT and APP/PS1 transgenic mice, respectively. LTGFβ1 (2.5 ng/mL) was added to the culture medium before PBMT (*n* = 3 per group). **d** Representative Western blotting assays for detecting the expression of newborn neuron-associated protein, Dcx/Tuj1, and astrocyte associated protein, GFAP, in vitro. **e** Quantification of Western blotting analysis of **d** (*n* = 3 per group). All quantifications are presented as mean ± SEM and were analyzed by one-way ANOVA test; **p < 0.01 versus control group; ^##^*p* < 0.01, ^#^*p* < 0.05 versus indicated group

First of all, we detected the generation of ROS in APP/PS1 NSCs after PBMT via flow cytometry and confocal imaging. The data showed that PBMT significantly increased ROS generation compared with that of the control group, whereas incubation with a ROS scavenger, NAC, reduced PBMT-induced ROS generation in APP/PS1 NSCs (Fig. 5a, b). Afterwards, we used enzyme-linked immunosorbent assays (ELISAs) to detect that the level of LTGFβ1 activated by PBMT increased by about 80% (Fig. 5c, *p* < 0.01) in vitro compared with the control group. Finally, we also detected the differentiation of APP/PS1 NSCs into neurons (Dcx^+^, Tuj1^+^ as markers) and astrocytes (GFAP^+^ as marker) under each experimental condition. Interestingly, the data showed that PBMT-treated NSCs exhibited the upregulation of Dcx/Tuj1 expression respectively by 31% and 75% (Fig. 5d, e, *p* < 0.01), while after incubation with NAC, the upregulation of Dcx/Tuj1 induced by PBMT was abolished. Notably, PBMT downregulated the expression of GFAP by 25% (Fig. 5d, e, Fig. S2a, *p* < 0.01). Taken together, these results suggest that APP/PS1 NSCs generated ROS to activate LTGFβ1, which represent an important condition for PBMT-induced APP/PS1 NSC differentiation to promote neurogenesis.

### PBMT-activated Smad2/3 interacts with Smad4 and competitively inhibits the Smad1/5/9 signaling pathway in APP/PS1 NSCs

**Fig. 6:**
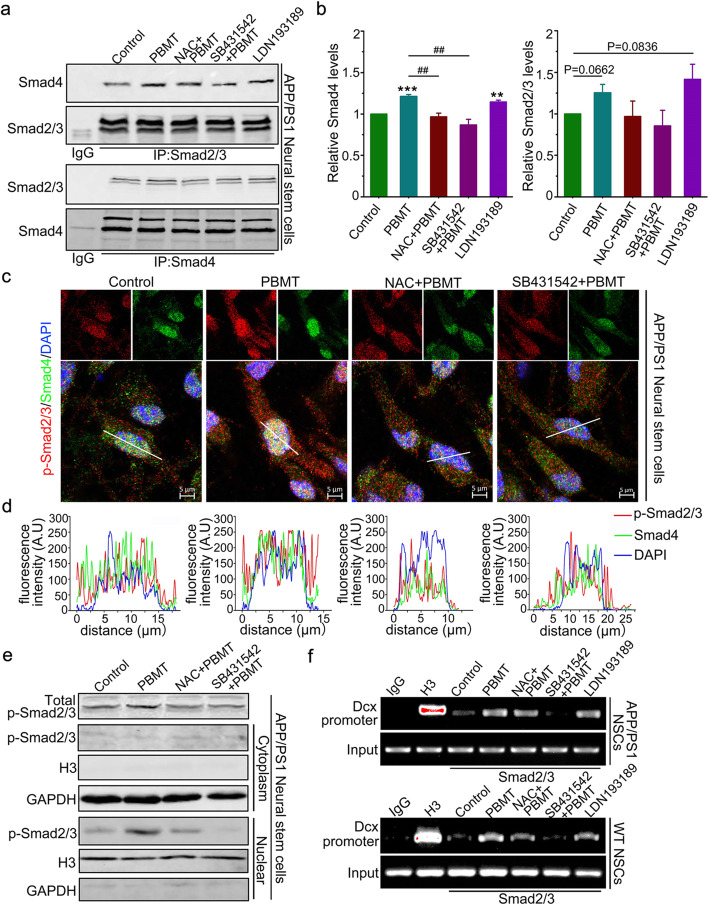
Activation of TGFβ1-Smad2/3 signaling by PBMT directs APP/PS1 NSC differentiation into neurons in vitro**. a** Co-immunoprecipitation with antibody to Smad2/3 and Smad4 in APP/PS1 NSCs, after co-incubation of NAC and SB431542 and LDN193189 (0.5 μM) before PBMT, the Western blotting analysis of the indicated proteins are shown. **b** Quantification of Smad4 and Smad2/3 relative levels for **a** (*n* = 3 per group). **c**, **d** Representative images (**c**) and fluorescence colocalization analysis (**d**) of co-translocation to the nucleus of p-Smad2/3 and Smad4 after PBMT, some groups have added the NAC and SB431542 before PBMT. Scale bar, 5 μm. **e** Western blotting analysis of nuclear cytoplasmic separation in APP/PS1 NSCs. Total extracts, cytosolic extracts, and nuclear extracts were immunoblotted for p-Smad2/3. Cytosolic and nuclear extracts were immunoblotted for GAPDH and Histone H3 protein to verify complete separation of the cytosolic and nuclear fraction. **f** Representative images and quantification the interaction of p-Smad2/3 in the chromatin environment of WT NSCs and APP/PS1 NSCs. Chromatin immunoprecipitation assays were performed as described in “Methods.” All quantifications are presented as mean ± SEM and were analyzed by one-way ANOVA test; ***p < 0.001, **p < 0.01 versus control group; ^##^*p* < 0.01 versus indicated group

We detected the interaction between Smad2/3 and Smad4 in APP/PS1 NSCs incubated with a morphogenetic protein 4 (BMP4) receptor inhibitor (LDN193189). The data showed that LDN193189 enhanced the interaction between Smad2/3 and Smad4 by 12% compared with that of the control group in APP/PS1 NSCs (Fig. 6a, b, *p* < 0.01). And we found that PBMT and increased the affinity of Smad2/3 to Smad4 by 25% in WT NSCs (Fig. S3a-S3c, *p* < 0.01) and by 23% in APP/PS1 NSCs (Fig. 6a, b, *p* < 0.001), which was detected by co-immunoprecipitation assays. The binding of Smad2/3 to Smad4 in APP/PS1 NSCs preincubated with NAC or SB431542 before PBMT treatments was significantly lower compared with that in PBMT-treated WT (Fig. S3b, S3c, *p* < 0.01) and APP/PS1 NSCs (Fig. 6b, *p* < 0.01) respectively.

For further details, we used Airyscan detection and nuclear separation assays to detect the nuclear translocation of p-Smad2/3 and Smad4. Indeed, PBMT increased the phosphorylation level of Smad2/3 to promote its translocation into nucleus, and thus enhancing the interaction of p-Smad2/3 with Smad4 in the nucleus, this phenomenon was reduced by preincubating with NAC or SB431542 before PBMT (Fig. 6c–e). Moreover, the results in Fig. S3d showed that there was a competitive relationship between p-Smad2/3 and p-Smad1/5/9 binding with Smad4 in NSCs after PBMT, and the phosphorylation levels of Smad2/3 were increased by PBMT, whereas the phosphorylation levels of Smad2/3 were relatively declined after preincubation of NAC or SB431542 before PBMT. Additionally, Smad1/5/9 phosphorylation in each group was negatively correlated with Smad2/3 phosphorylation (Fig. S3d).

The quantitative real-time PCR results revealed that PBMT-treated NSCs upregulated the mRNA levels of Dcx by 90% (Fig. S4a, *p* < 0.001) and downregulated the mRNA levels of GFAP by 95% (Fig. S4a, *p* < 0.01). However, in NSCs preincubated with NAC or SB431542 before PBMT, Dcx mRNA levels were downregulated, while GFAP mRNA levels were upregulated (Fig. S4a, *p* < 0.001). The protein levels of Dcx/GFAP and the number of Dcx^+^/GFAP^+^ cells in APP/PS1 NSCs after PBMT-induced were detected via western blotting and immunofluorescence staining (Fig. S4b-S4d), the results are in line with Fig. S4a.

In addition, chromatin immunoprecipitation assays (the primer design method is shown in Fig. S5a-S5e) indicated that p-Smad2/3 binding to Smad4 acted as transcription factors during the PBMT-induced differentiation of WT NSCs and APP/PS1 NSCs into neurons and had a direct effect on transcriptional regulation of the newborn neuron-associated protein, Dcx, in vitro (Fig. 6f).

### Activation of TGFβ1-Smad2/3 signaling pathway by PBMT interacts with Smad4, upregulates Dcx expression, and competitively inhibits GFAP expression in APP/PS1 mice

**Fig. 7:**
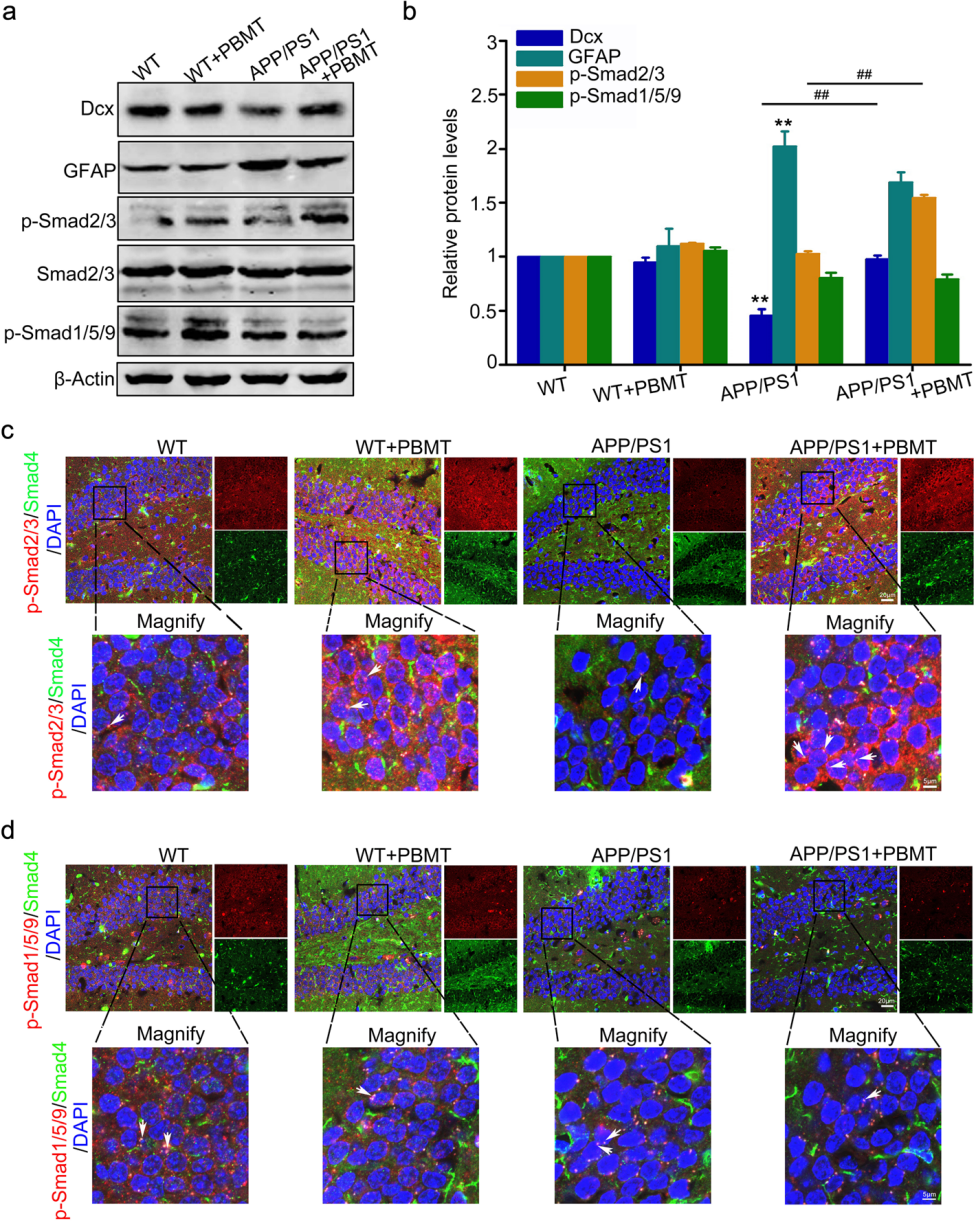
PBMT-activated Smad2/3 interacts with Smad4 and upregulates Dcx expression and competitively inhibit GFAP expression in APP/PS1 mice**. a** Western blotting analyzes the Dcx, GFAP, p-Smad2/3, and p-Smad1/5/9 protein expression levels of WT and APP/PS1 mice after PBMT. **b** Statistical analysis results of Dcx and GFAP protein expression in hippocampus of WT and APP/PS1 mice after PBMT 1 month, and phosphorylation levels of Smad2/3 and Smad1/5/9 within 30 min after PBMT (*n* = 4 per group). **c**, **d** Representative images of colocalization of p-Smad2/3 with Smad4 (**c**) and representative images of colocalization of p-Smad1/5/9 with Smad4 (**d**) in brain tissue of WT and APP/PS1 mice after PBMT 1 month. Scale bar, 20 μm (the magnify image scale bar is 5 μm). All quantifications are presented as mean ± SEM and were analyzed by one-way ANOVA test; **p < 0.01 versus control group; ^##^*p* < 0.01 versus indicated group

To further explore the activation of TGFβ1-Smad2/3 signal in vivo, we detected Smad2/3 phosphorylation and Dcx expression in tissues from APP/PS1 mice via western blotting. The results showed that PBMT-treated APP/PS1 mice exhibited the increase of p-Smad2/3 by 51% (Fig. 7a, b, *p* < 0.01), PBMT treatment upregulated the expression of Dcx by 46% and downregulated the expression of GFAP by 35% (Fig. 7b, *p* < 0.01). Moreover, we found that in the hippocampus of APP/PS1 mice, phosphorylation of Smad2/3 and interaction with Smad4 were increased after PBMT (Fig. 7c), while the interaction between p-Smad1/5/9 and Smad4 relatively declined (Fig. 7d). These in vivo results were in line with in vitro results (Fig. S6a-S6d).

## Discussion

**Fig. 8:**
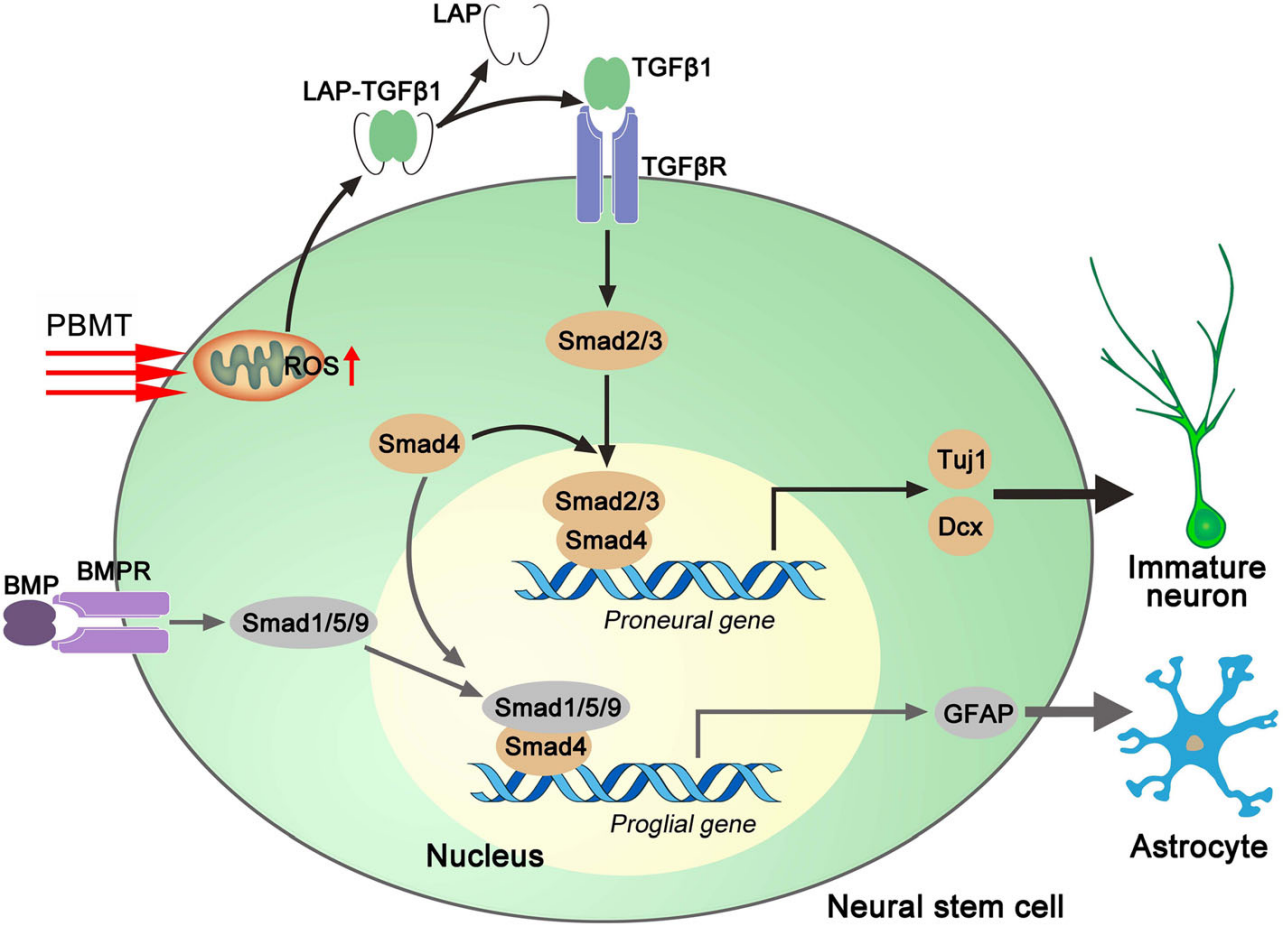
Schematic representation for photoactivation of TGFβ/SMAD signaling pathway ameliorates adult hippocampal neurogenesis in Alzheimer’s disease model

In our study, we demonstrated that PBMT directed APP/PS1 NSCs to differentiate into neurons and reduced differentiation into astrocytes. The mechanisms underlying this process involved was PBMT generating ROS in APP/PS1 NSCs, which activated LTGFβ1 to induce TGFβ1 promoting Smad2/3 phosphorylation and competition with p-Smad1/5/9 for Smad4 binding. Subsequently, complexes of p-Smad2/3 binding to Smad4 serve as Dcx transcription factors to promote Dcx transcription, and directed NSC differentiation into neurons to induce AHN in APP/PS1 mice (Fig. 8). The normal expression patterns of functional proteins in neuron—such as AMPARs, PSD-95, and MAP2—directly affect the function of neurons and influence the integrities of both human and animal nervous systems, as well as influence neurological activities, such as thinking, consciousness, and emotion [45, 46]. In addition, neuronal apoptosis and neurogenesis in the hippocampus have significant effects on cognitive behavior in mice [47]. We found that PBMT-induced newborn neurons expressed neuronal function-related proteins (MAP2, AMPARs, PSD-95) and PBMT effectively improves spatial learning/memory in APP/PS1 mice, implying that these newborn neurons may be involved in the transduction and signal transmission of neural circuits. Therefore, our findings suggest that PBMT may represent a viable means to promote AHN to ameliorate cognitive impairment and behavioral abnormalities caused by AD pathology.

Newborn neurons convey specific types of neuroplasticity, and glial cells modulate neuronal function which have been increasingly appreciated [48, 49]. The thickness of myelin is dynamically modulated based on experience, and the generation of new oligodendrocytes is required in the mouse brain to learn certain new tasks [50–52]; the above research results suggest that oligodendrocyte generation on-demand increased myelination for active neural circuits [53–56]. AD neuropathological hallmarks include extracellular Aβ plaques and intracellular tau-hyperphosphorylated NFTs [57], which are non-homeostasis that restrict neurogenesis and gliogenesis. NSC transplantation into the hippocampus gives rise to all three cellular lineages (neurons, astrocytes, and oligodendrocytes) to not only replace missing tissue but also produce a variety of cytokines, promote synapse formation, and regulate synaptic plasticity [40, 58], all of which contribute to ameliorating cognitive, learning, and memory deficits in mouse models of AD. However, whether NSCs and glial cell differentiation replace injured nerve tissue, or if cytokines secreted by endogenous NSCs or glial cells activate certain signaling pathways to promote neurogenesis and gliogenesis, remains controversial. In the present study, our in vivo results demonstrated that PBMT not only directed the differentiation of endogenous APP/PS1 NSCs into neurons, but also promoted gliogenesis. Both newborn neurons and glial cells may have several specific contributions to neural plasticity and to neurodegenerative repair in AD mouse brain after PBMT.

Members of the TGFβ superfamily play a critical role in brain development [59, 60], TGFβ signal transduction contributes to maintaining stem cells in a resting phase and to promoting survival and functional differentiation of newborn neurons [33]. Meanwhile, several reports have indicated that BMP4-mediated downstream signaling pathway promotes the differentiation of NSCs into astrocytes [61, 62], and spinal neurogenesis is promoted by inhibition of BMP signaling via expression of Noggin [63]. Furthermore, TGFβ and Smad4 mutant mice exhibit highly increased neuronal apoptosis [64, 65]. In contrast, previous studies have shown that when the TGFβ1-Smad2/3 signaling pathway is inhibited, NSCs differentiate more into astrocytes than into neurons. In neural precursor cells, TGFβ exhibits differential biological effects at different stages of development, and this phenomenon may be due to latency-associated peptide (LAP)-TGFβ1 (LTGFβ1) being activated, such that TGFβ1 then further activates the downstream signaling pathways [66]. Cytochrome c oxidase (CcO) is a critical enzyme that catalyzes the final step in the mitochondrial electron transport chain, and CcO has been widely considered to be a photoreceptor of PBMT, and transient cytosolic ATP or ROS increase was a photobiological consequence by PBMT via acting CcO [28, 67–71]. The generation of small amount of ROS, as natural messengers, causes intracellular signaling cascades to regulate cell activity [72]. Notably, the cells derived from different classifications have different responses upon PBMT-induced ROS; these specific biological effects upon PBMT-induced ROS include promotion of proliferation and differentiation, regulation of immune/inflammation, expression of transcription factors and cytokines, which are very important for neurology, dentology, dermatology, and regenerative medicine [69]. The LTGFβ1 has a specific methionine (position 253, which confers reactive oxygen species [ROS] sensitivity) on LAP, and this specific methionine can be photoactivated [73]. In this study, as a viable noninvasive treatment, PBMT can promote the generation of ROS in APP/PS1 NSCs within neurogenic niches, which activate LAP-TGFβ1 thereby activating the TGFβ1-Smad2/3 signaling pathway to induce differentiation of APP/PS1 NSCs into neurons while inhibiting differentiation into astrocyte. Meanwhile, our data revealed that the competitive binding of p-Smad2/3 to Smad4 and p-Smad1/5/9 to Smad4 in NSCs was a key mechanism under PBMT. Thus, we found that Smad4 was a key link in PBMT-mediated neurogenesis under AD-like pathological conditions.

There is a brief increase in ROS generated in the mitochondria when they absorb the photons delivered during PBMT treatment, and this burst of ROS will trigger some mitochondrial signaling pathways leading to cytoprotective, antioxidant, and antiapoptotic effects in the cells [69, 74]. In terms of antioxidation, there are multiple antioxidant enzymes in cell function to scavenge and degrade ROS in different subcellular compartments to allow the development and persistence of oxidative signals [75, 76]. Moreover, the antioxidant system helps regulate ROS signaling by limiting the extent of protein oxidation in both spatial and time [75]. In other cases, antioxidant enzymes, acting as a redox relay, transmit oxidant signals from ROS to selective protein targets [77]. For example, in *Saccharomyces cerevisiae*, glutathione peroxidase (Gpx3) act as a redox relay that transduces H_2_O_2_ to oxidatively activate the transcription factor Yap1 [75, 78]. Therefore, because of the existence of the antioxidant system in diverse biological systems, the ROS signaling was refined and enhanced rather than prevented in redox signaling [74, 79]. Although the occurrence of cell oxidative stress under low-dose PBMT treatment has not been detected so far, the more detailed antioxidant mechanism needs further exploration. In addition, PBMT suppressed the expression of several inflammatory cytokines, such as proinflammatory gene interleukin-1 beta (IL-1β) and tumor necrosis factor alpha (TNF-α) [69, 72]. It has been reported that PBMT increased the activity of microglia, promoted the removal of Aβ plaques, and reduced the inflammation in the brain of AD mice, thereby alleviating AD symptoms [27, 69, 80]. Hence, these findings reflected the antiinflammation of PBMT.

Adult neurogenesis declines in an age-dependent manner, which may partially underlie cognitive impairment in the elderly. The microenvironmental homeostasis in NSC niches is disturbed under AD pathological conditions and inhibition of neurogenesis may be one of the mechanisms responsible for AD-related cognitive deficits [8, 19, 81]. In this study, PBMT-activated NSCs from a quiescent state, to increase proliferation and differentiation, even under AD-like pathological conditions. Furthermore, our previous work has shown that PBMT can increase the expression of BDNF and rescue dendritic atrophy in the hippocampus of APP/PS1 neurons [26]. In combination with the increase in neurotrophic factors, the differentiation of NSCs into neurons under PBMT in this study suggests that PBMT may provide a potential treatment to improve the efficiency of neurogenesis in AD and elderly conditions and ameliorate neurodegenerative phenomena.

## Conclusions

Our research demonstrates that PBMT, as a novel noninvasive physiotherapy strategy, promotes the differentiation of APP/PS1 NSCs into neurons and reduces the differentiation into astrocytes by activating TGFβ/Smad signaling pathway. Importantly, our study shows that PBMT improved the spatial learning/memory ability in APP/PS1 mice, which may be correlated with an increase in the number of newborn hippocampal neurons. Collectively, the in vitro and in vivo results suggest that PBMT has great potential therapeutic value for ameliorating the drop of AHN in Alzheimer’s disease mice.

## Supplementary Information

**Additional file 1.**

## Data Availability

Raw data is available from the corresponding author upon reasonable request.

## Abbreviations

PBMT: Photobiomodulation therapy
AD: Alzheimer’s disease
AHN: Adult hippocampal neurogenesis
APP/PS1: Amyloid precursor protein/presenilin 1
WT: Wild-type
LTGFβ1: Latent transforming growth factor-β1
NSCs: Neural stem cells
Dcx: Doublecortin
Tuj1: Neuronal class-III β-tubulin
ROS: Reactive oxygen species
TGFβR: Transforming growth factorβ receptor
BMP: Bone morphogenetic protein
GFAP: Glial fibrillary acidic protein
TGFβR I: TGFβ receptor I
SGZ: Subgranular zone
SVZ: Subventricular zone
GCL: Granule cell layer
Aβ: Amyloid-β
NFTs: Neurofibrillary tangles
SP: Senile plaque
CNS: Central nervous system
AMPARs: α-Amino-3-hydroxy-5-methyl-4-isoxazole-propionic acid receptors
MAP 2: Microtubule-associated protein 2
PSD-95: Postsynaptic density protein 95
NAC: N-acetyl cysteine
MWM: Morris water maze
CcO: Cytochrome c oxidase
Gpx3: Glutathione peroxidase
IL-1β: Interleukin-1 beta
TNF-α: Tumor necrosis factor alpha
