# Supplementary material for: Photoactivation of TGFβ/SMAD signaling pathway ameliorates adult hippocampal neurogenesis in Alzheimer’s disease model
Source: Stem Cell Res Ther. 2021 Jun 11;12:345. doi: 10.1186/s13287-021-02399-2 (PMC8196501; doi:10.1186/s13287-021-02399-2)
Supplement: Supplementary file 1 — Additional file 1. [file 13287_2021_2399_MOESM1_ESM.doc]

**Supplemental materials**

**Supplemental figures**


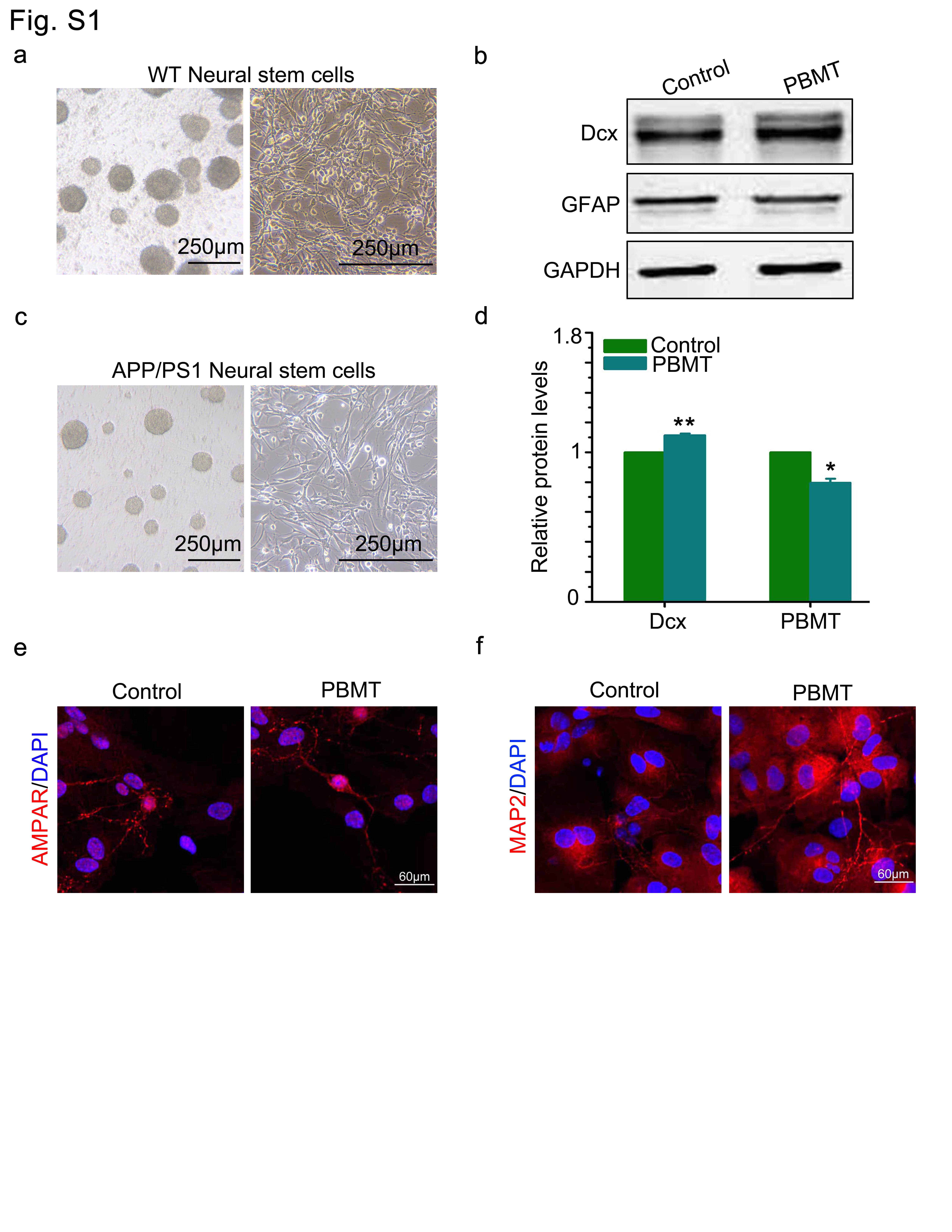


**Fig. S1 Detection of neural stem cells (NSCs) differentiation and the expression of α-amino-3-hydroxy-5-methyl-4-isoxazole-propionic acid receptors (AMPAR)/** **microtubule- associated protein 2 (MAP2) of newborn neurons after photobiomodulation therapy (PBMT) *in vitro*.**

**a** and **b**, State record after 3 days of suspension culture of NSCs from wild-type (WT) **(a)** and amyloid precursor protein/presenilin 1 (APP/PS1) **(b)** fetal mouse (E14 d) hippocampus and 3 days after first passage of adherent culture (with 0.6% Matrigel as supporting). Scale bar: 250 μm. **c** and **d**, Western blotting analysis **(c)** and quantification **(d)** the expression of doublecortin (Dcx) and glial fibrillary acidic protein (GFAP) in WT NSCs after PBMT induced differentiation *in vitro*, (*n* = 4 per group). **e** and **f**, Representative images of immunofluorescence of neuron-associated functional protein AMPAR **(e)** and MAP2 **(f)** expression from newborn neurons by NSCs differentiation *in vitro*. Scale bar, 60 μm. All quantifications are presented as mean ± SEM and were analyzed by student’s *t* tests; ***p* < 0.01, **p* < 0.05 versus control group.

**
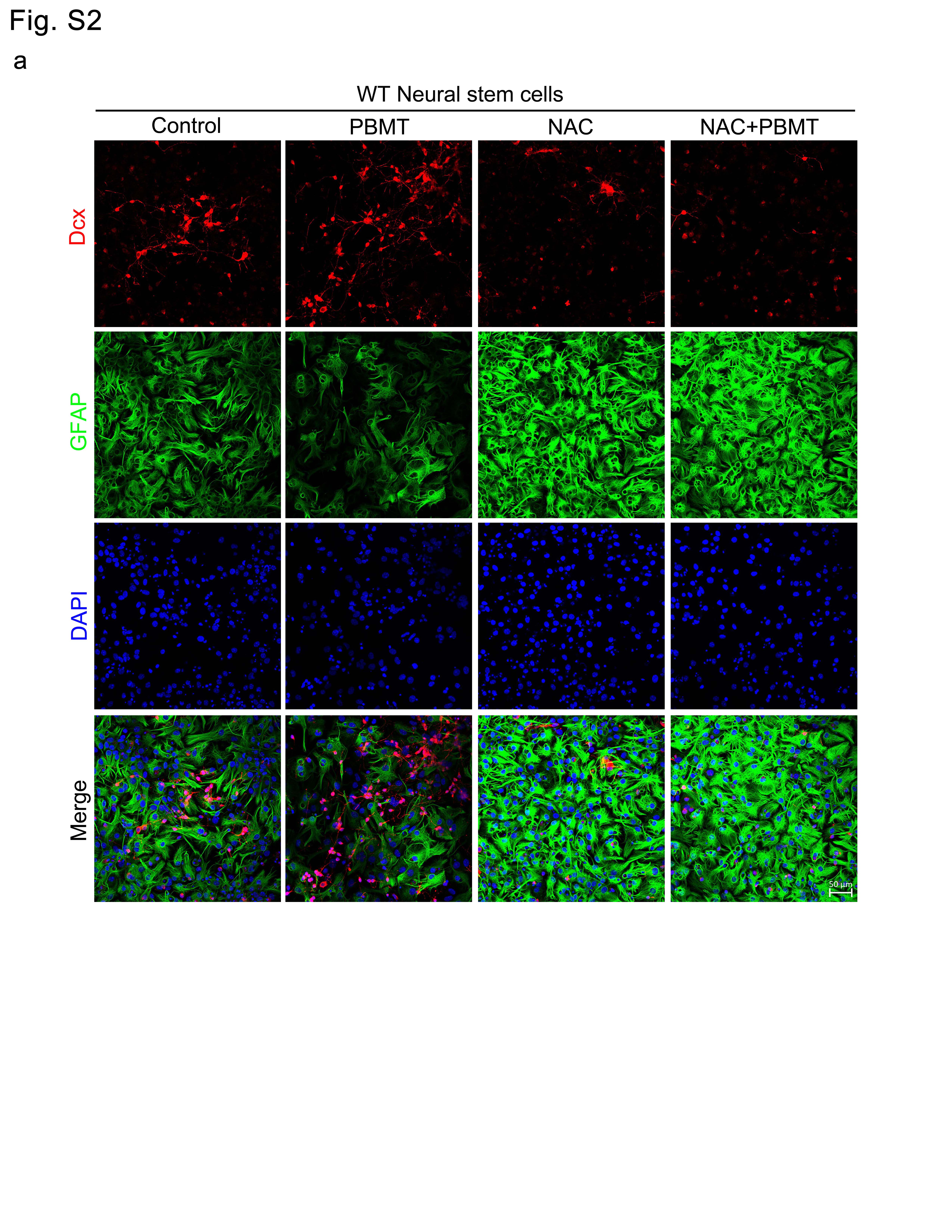
**

**Fig. S2 Elimination of** **reactive oxygen species (ROS) decreased WT NSCs differentiation after PBMT *in vitro*.**

**a**, Representative immunofluorescence images of WT NSCs differentiation induced by PBMT *in vitro*. Some groups were preincubated with NAC (1 mM) before PBMT. Cell lineage markers used were Dcx for newborn neurons, GFAP for astrocytes. Scale bar, 50 μm.


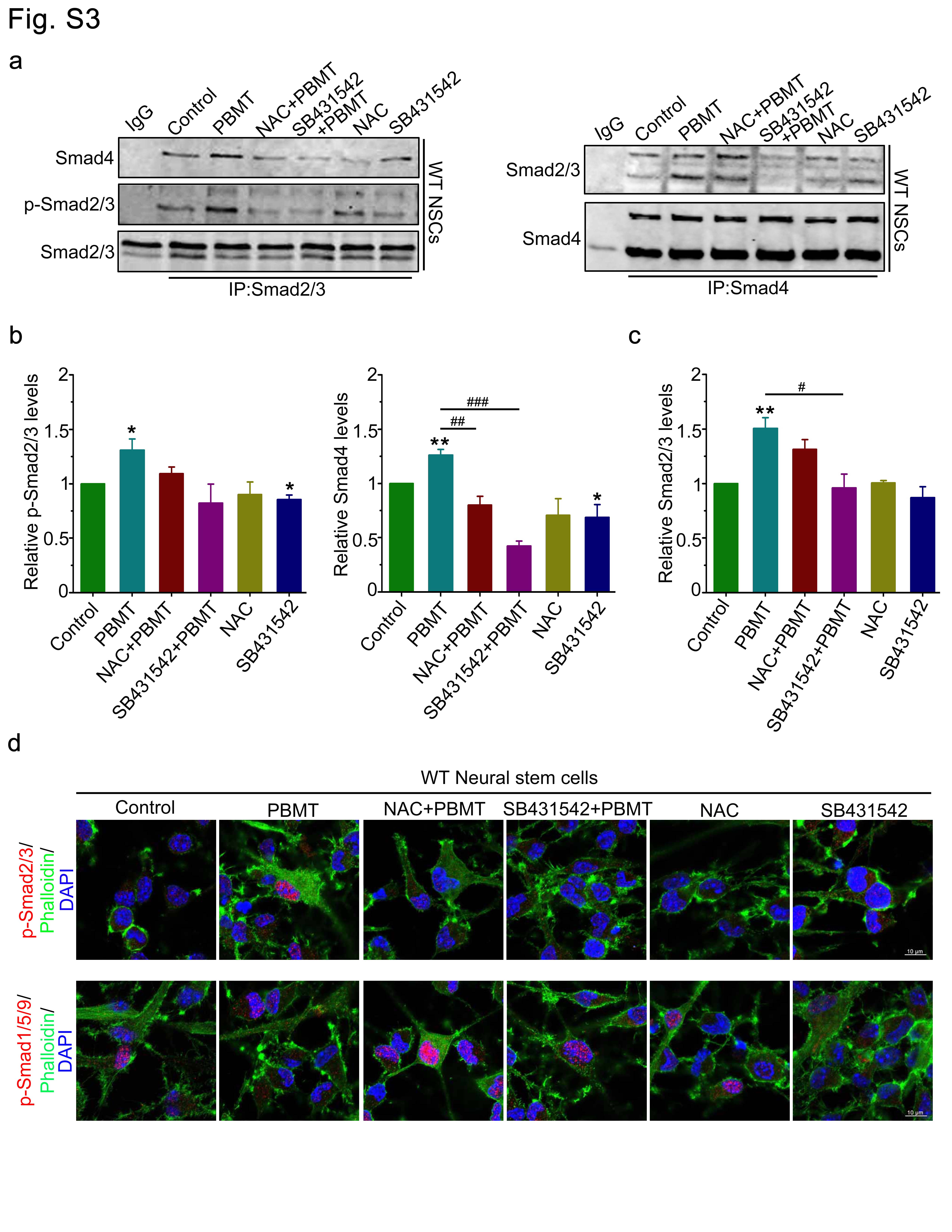


**Fig. S3 PBMT activates TGFβ-Smad2/3 signaling and competitively inhibits BMP-Smad1/5/9 signaling in WT NSCs *in vitro*.**

**a**, Immunoprecipitation with antibody to Smad2/3 or Smad4 in WT NSCs, after co-incubation of NAC and SB431542 before PBMT, the Western blotting analysis of the indicated proteins are shown. IgG, immunoglobulin G. **b** and **c**, Quantification of p-Smad2/3, Smad4 **(b)** and Smad2/3 **(c)** relative levels for **a**, (*n* = 3 per group). **d**, Representative immunofluorescence images of phosphorylation levels of Smad2/3 and Smad1/5/9, the TGFβ1 responsiveness of NSCs was assessed by p-Smad2/3 translocate to nucleus after PBMT, the bone morphogenetic protein (BMP) responsiveness of NSCs was assessed by p-Smad1/5/9 translocate to nucleus. Phalloidin lining the cytoskeleton, DAPI [4′,6-diamidino-2-phenylindole] staining the nucleus. Tricolor merge are shown. Some cells were preincubated with NAC or SB431542 before PBMT. Scale bar, 10 μm. All quantifications are presented as mean ± SEM and were analyzed by One-way ANOVA test; ***p* < 0.01, **p* < 0.05 versus control group; ^###^*p* < 0.001, ^##^*p* < 0.01, ^#^*p* < 0.05 versus indicated group.

**
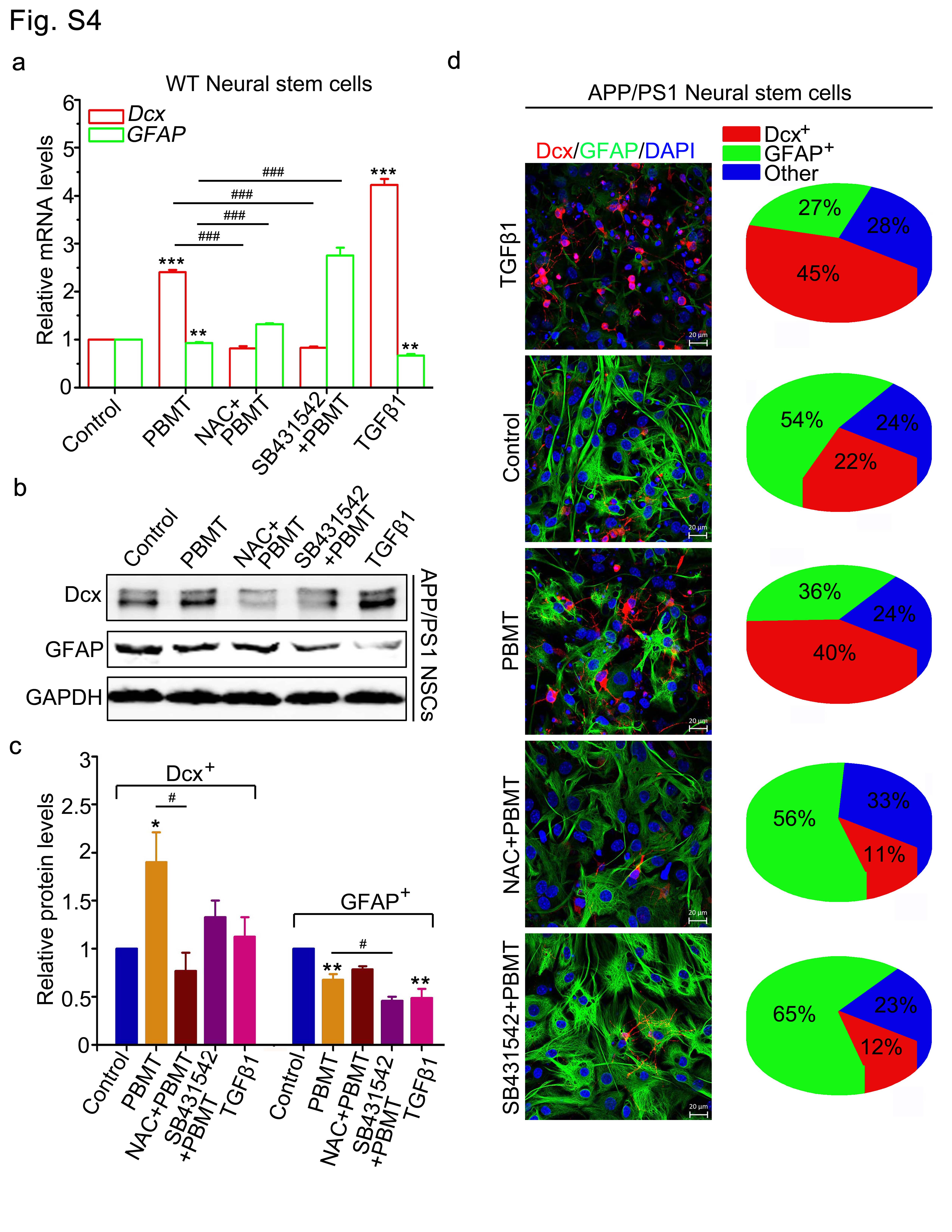
**

**Fig. S4 Activation of TGFβ-Smad2/3 signal pathway by PBMT directs NSCs to differentiate into neurons and to reduce NSCs differentiate into astrocytes *in vitro*.**

**a**, Detection of *dcx* , *gfap* gene transcription levels during differentiation of WT NSCs by real-time PCR after PBMT. Some group cells were incubated with NAC (1 mM) and SB431542 (60 μM) one hour before PBMT, TGFβ1 (2 ng/mL) group as a positive control, total RNA extracted 8 hours after PBMT, (*n* = 5 per group). **b** and **c**, Western blotting analysis **(b)** and quantification **(c)** of APP/PS1 NSCs differentiation induced by PBMT *in vitro*, the processing group in this part is in line with **a**, (*n* = 3 per group). **d**, The differentiation of APP/PS1 NSCs after PBMT, some cells were pretreated with SB431542 (60 μM) or NAC (1 mM) before PBMT, TGFβ1 treatment group as a positive control. Scale bar, 20 μm. All quantifications are presented as mean ± SEM and were analyzed by One-Way ANOVA test; ****p* < 0.001, ***p* < 0.01, **p* < 0.05 versus control group; ^###^*p* < 0.001, ^#^*p* < 0.05 versus indicated group.


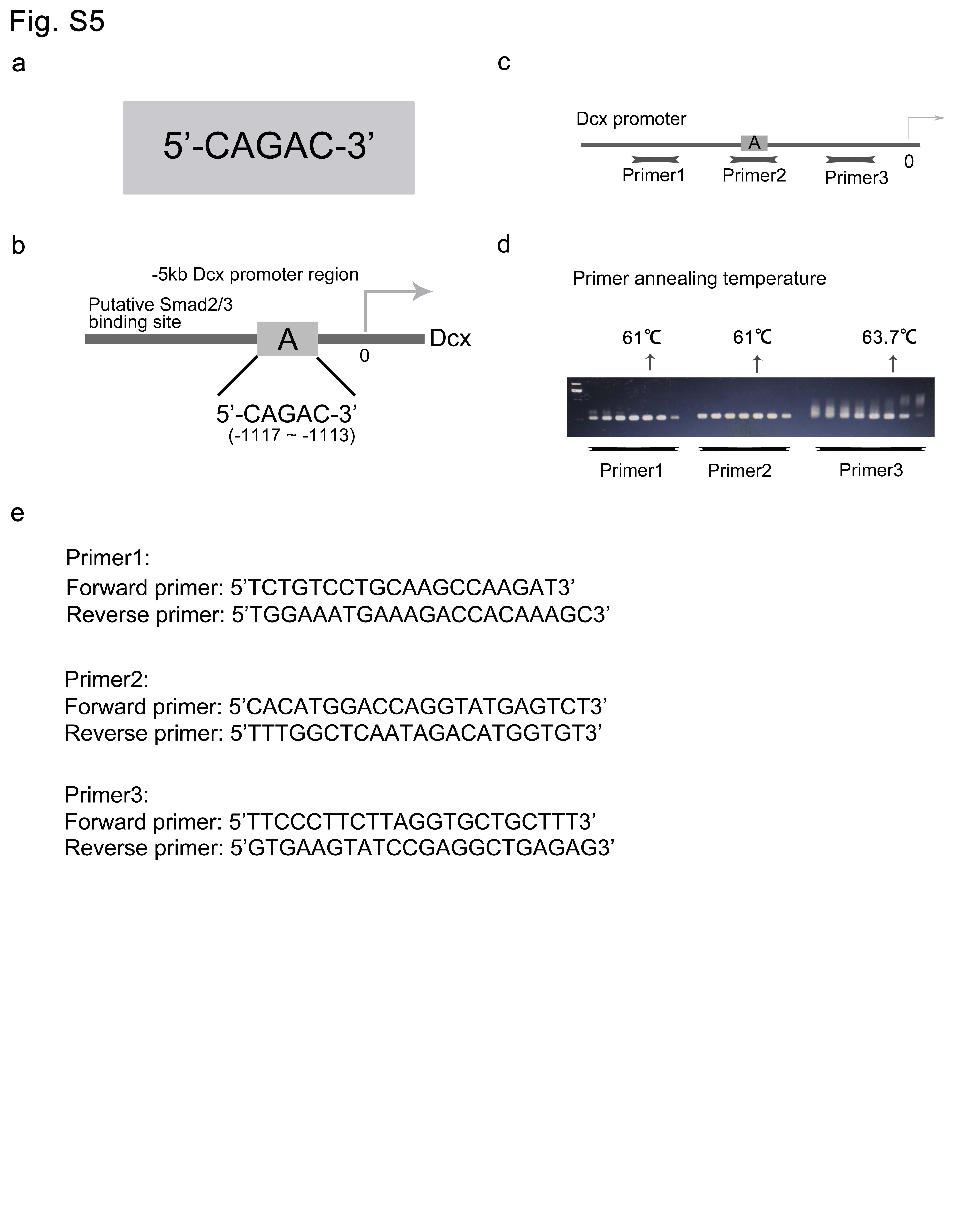


**Fig. S5** Chromatin immunoprecipitation primer design process.

**a**, The canonical binding motifs of mouse *Dcx* gene promoter. **b**, One Smad2/3 putative binding sites in the *Dcx* promoter. **c**, Design primers for the upstream of the A site, including the A site and the A site downstream promoter region. **d** and **e**, Annealing temperature determination **(d)** and primer sequence **(e)**.


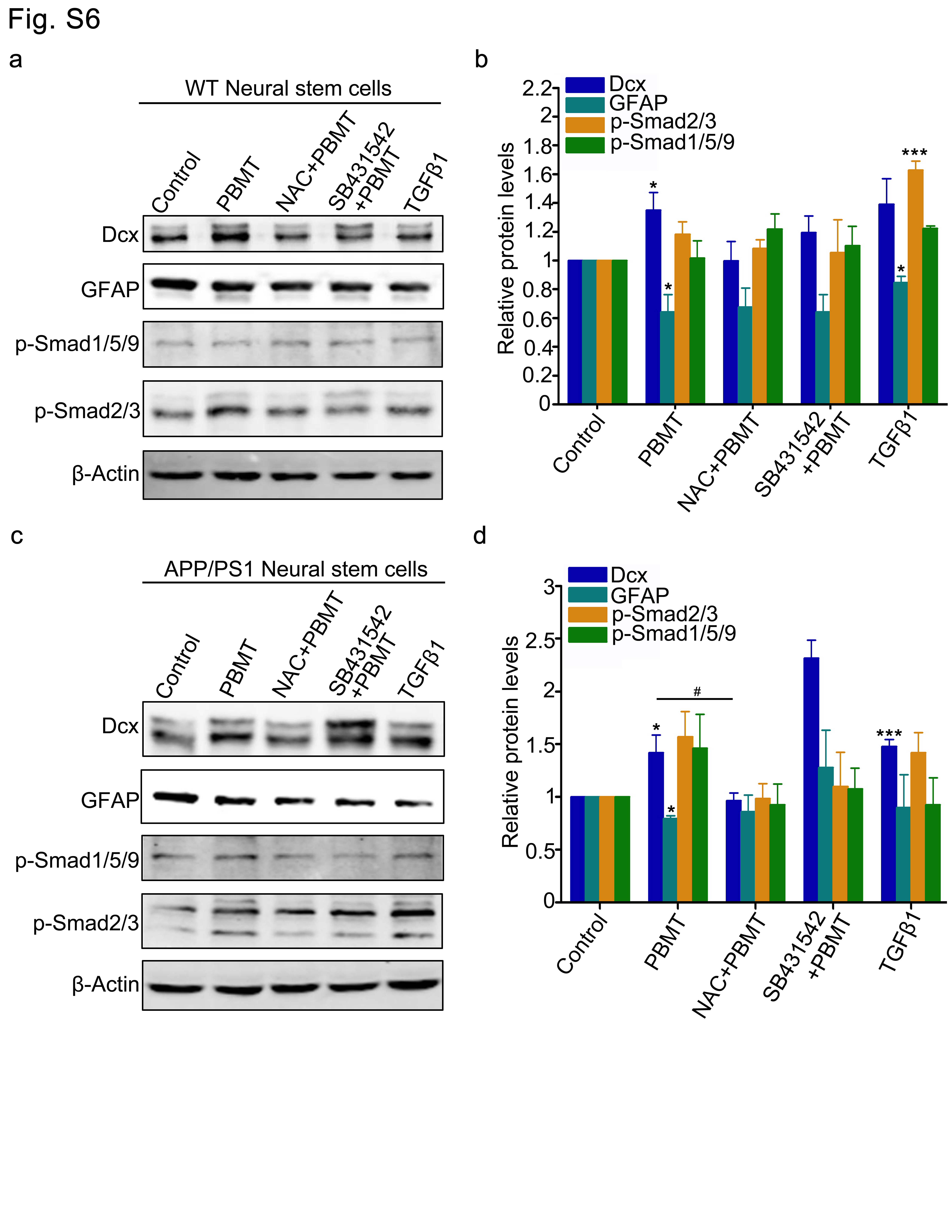


**Fig. S6 PBMT guides both and APP/PS1 NSCs differentiation by activating TGFβ/Smad signaling pathway *in vitro*.**

**a** and **b**, Western blotting analysis **(a)** and quantification **(b)** of Dcx and GFAP protein expression levels and Smad2/3 and Smad1/5/9 phosphorylation levels of WT NSCs after PBMT, some group cells were incubated with NAC (1 mM) and SB431542 (60 μM) one hour before PBMT detection of phosphorylation levels is detected within 30 minutes after PBMT, TGFβ1 (2 ng/mL) group as a positive control to promote the differentiation of NSCs into neurons. (*n* = 3 per group). **c** and **d**, Western blotting analysis **(c)** and quantification **(d)** of Dcx and GFAP protein expression levels and Smad2/3 and Smad1/5/9 phosphorylation levels of APP/PS1 NSCs after PBMT, the processing mode and detection time of each group are in line with those in **a** and **b**. (*n* = 3 per group). All quantifications are presented as mean ± SEM and were analyzed by One-way ANOVA test; ****p* < 0.001, **p* < 0.05 versus control group; ^#^*p* < 0.05 versus indicated group.

**Tables S1** Key resources table

| Reagent or Resource | | Source | | | | Identifier |
| --- | --- | --- | --- | --- | --- | --- |
| Antibodies | | | | | | |
| Rabbit anti-DCX (1:250) | | Abcam | | | | Cat#ab207175 |
| Rabbit anti-Tuj1 (1:100) | | Proteintech | | | | Cat#10068-1-AP;  RRID: AB_2303998 |
| Mouse anti-GFAP (1:300) | | Cell Signaling Technology | | | | Cat#3670;  RRID: AB_561049 |
| Mouse anti-Smad4 (1:50) | | Santa Cruz Biotechnology | | | | Cat#SC-7966;  RRID: AB_627905 |
| Rabbit anti-Smad2/3 (1:300) | | Cell Signaling Technology | | | | Cat#8685;  RRID: AB_10889933 |
| Rabbit  anti-p-Smad2/3 (1:300) | | Cell Signaling Technology | | | | Cat#8828;  RRID: AB_2631089 |
| Rabbit  anti-p-Smad1/5/9 (1:300) | | Cell Signaling Technology | | | | Cat#13820;  RRID: AB_2493181 |
| Goat anti-Mouse 488 (1:400) | | Abcam | | | | Cat#ab150113;  RRID: AB_2576208 |
| Goat anti-Rabbit 555 (1:400) | | Abcam | | | | Cat#ab150078;  RRID: AB_2722519 |
| Goat  anti-Mouse 680 (1:10000) | | Abcam | | | | Cat#ab175775 |
| Goat  anti-Rabbit 790 (1:15000) | | Abcam | | | | Cat#ab175781 |
| Chemicals, Peptides, and Recombinant Proteins | | | | | | |
| Fetal bovine serum | | GIBCO | | | | Cat#10270 |
| DMEM/F-12 | | GIBCO | | | | Cat#11330-032 |
| B27 | | GIBCO | | | | Cat#17504-044 |
| Human EGF | | GIBCO | | | | Cat#PHG0314 |
| Human FGF-basic | | GIBCO | | | | Cat#13256-029 |
| Human Latent TGF-β1 | | R&D Systems | | | | Cat#299-LT-005 |
| Human TGF-β1 | | PeproTech | | | | Cat#100-21C |
| NeuroCult  Differentiation Medium | | Stem Cell Technologies | | | | Cat#05704 |
| StemPro Auccutase | | GIBCO | | | | Cat#A11105-01 |
| Trypsin-EDTA (0.05%) | | GIBCO | | | | Cat#25300054 |
| Protease inhibitor Cocktail | | Roche | | | | Cat#5892791001 |
| HBSS(10X) | | GENMED | | | | Cat#GMS12033.2 |
| DPBS | | GIBCO | | | | Cat#14190-136 |
| Matrigel | | BD | | | | Cat#356234 |
| Phalloidin-FITC | | Beyotime | | | | Cat#C1033 |
|  | |  | | | | *(Continued on next page)* |
| *Continued* | | | | | | |
| Reagent or Resource | | | Source | | Identifier | |
| SB431542 | | | MCE | | Cat#HY-10431 | |
| LDN193189 | | | MCE | | Cat#HY-12071A | |
| SYBR Green PreMix | | | Takara | | Cat#RR820A | |
| DAPI for nucleic acid staining | | | Sigma | | Cat#D9542-1MG | |
| Critical Commercial Assays | | | | | | |
| Reactive oxygen species assay kit | | | Beyotime | | Cat#S0033 | |
| TGFβ1 E_max_ Immuno Assay System | | | Promega | | Cat#G7590 | |
| Simple ChIP Enzymatic Chromatin IP Kit | | | Cell Signaling Technologies | | Cat#91820 | |
| Nuclear separation kit | | | BestBio | | Cat#BB3102 | |
| Experimental Models: Primary Cells | | | | | | |
| WT mice hippocampal neural stem cell | | | This paper | | N/A | |
| APP/PS1 mice hippocampal neural stem cell | | | This paper | | N/A | |
| Experimental Models: Organisms | | | | | | |
| Mouse: C57BL/6J | | | | | | |
| Mouse: APP/PS1 Transgenic | | | | | | |
| Oligonucleotides | | | | | | |
| GFAP-Fw: AATGCTGGCTTCAAGGAGAC | | | Lifetech | | N/A | |
| GFAP-Rv: AAGCGGACCTTCTCGATGTA | | | Lifetech | | N/A | |
| Dcx-Fw: GTGCTCAAGCCAGAGAGAACAA | | | Lifetech | | N/A | |
| Dcx-Rv: ACCCCGCTGCGAATGAT | | | Lifetech | | N/A | |
| Software and Algorithms | | | | | | |
| LSM 880 Confocal | | | Zeiss | | N/A | |
| Image J | | | National Institute of Health | | RRID: SCR_003070;  https://imagej.net/Welcome | |
| Odesseys | | | LI-COR | | http://biosupport.licor.com | |
| Origin | | | Microcal Software | | https://www.microcal.com | |
| ZEN digital imaging for  light microscopy | | | Zeiss | | https://www.zeiss.com/  microscopy/ us/products/  microscopesoftware/zen.html | |
| Other | | | | | | |
| Orifice plate | Corning | | | Cat#3516 | | |

**Tables S2** laser parameters used *in vivo*

| Light source Semiconductor laser |  | Semiconductor laser |
| --- | --- | --- |
| Center wavelength (nm) 635 nm | | |
| Beam spot size at target (cm^2^) |  | 0.785 cm^2^ |
| Exposure duration (sec) |  | 600 sec |
| Radiant exposure (J/cm^2^) |  | 2 J/cm^2^ |
| Radiant energy (J) |  | 1.57 J |
| Number of points irradiated |  | 1 |
| Application technique |  | Without skin contact |
| Number of treatment sessions |  | 30 sessions |
| Frequency of treatment sessions |  | Once a day |
| Total radiant energy (J) |  | 1 J per session, 47.1 J over all sessions |

**Tables S3** laser parameters used *in vitro*

| Light source Semiconductor laser |  | Semiconductor laser |
| --- | --- | --- |
| Center wavelength (nm) 635 nm | | |
| Beam spot size at target (cm^2^) |  | 9.6 cm^2^ |
| Exposure duration (sec) |  | 150 sec |
| Radiant exposure (J/cm^2^) |  | 2 J/cm^2^ |
| Radiant energy (J) |  | 19.2 J |
| Number of points irradiated |  | 1 |
| Application technique |  | Without surface contact |
| Number of treatment sessions |  | 1 sessions |
